# Susceptibility gene mutations in germline and tumors of patients with HER2-negative advanced breast cancer

**DOI:** 10.1038/s41523-024-00667-x

**Published:** 2024-07-13

**Authors:** Peter A. Fasching, Chunling Hu, Steven N. Hart, Matthias Ruebner, Eric C. Polley, Rohan D. Gnanaolivu, Andreas D. Hartkopf, Hanna Huebner, Wolfgang Janni, Peyman Hadji, Hans Tesch, Sabrina Uhrig, Johannes Ettl, Michael P. Lux, Diana Lüftner, Markus Wallwiener, Lena A. Wurmthaler, Chloë Goossens, Volkmar Müller, Matthias W. Beckmann, Alexander Hein, Daniel Anetsberger, Erik Belleville, Pauline Wimberger, Michael Untch, Arif B. Ekici, Hans-Christian Kolberg, Arndt Hartmann, Florin-Andrei Taran, Tanja N. Fehm, Diethelm Wallwiener, Sara Y. Brucker, Andreas Schneeweiss, Lothar Häberle, Fergus J. Couch

**Affiliations:** 1 Department of Gynecology and Obstetrics, Erlangen University Hospital, Comprehensive Cancer Center Erlangen-EMN, Friedrich-Alexander Universität Erlangen-Nürnberg, Erlangen, Germany; 2 Department of Laboratory Medicine and Pathology, Mayo Clinic, Rochester, MN USA; 3 Department of Quantitative Health Sciences, Mayo Clinic, Rochester, MN USA; 4 Department of Obstetrics and Gynecology, University of Tübingen, Tübingen, Germany; 5 Department of Gynecology and Obstetrics, Ulm University Hospital, Ulm, Germany; 6 Frankfurt Center for Bone Health, Frankfurt am Main, Germany; 7 Oncology Practice, Bethanien Hospital, Frankfurt am Main, Germany; 8 Department of Obstetrics and Gynecology, Klinikum rechts der Isar, Technical University of Munich, Munich, Germany; 9 Department of Gynecology and Obstetrics, Frauenklinik St. Louise, Paderborn, St. Josefs-Krankenhaus, Salzkotten, Germany; St. Vincenz Kliniken Salzkotten + Paderborn, Paderborn, Germany; 10 Immanuel Klinik Märkische Schweiz & Medical University of Brandenburg Theodor Fontane, Rüdersdorf bei Berlin, Buckow, Germany; 11 Department of Gynecology, Halle University Hospital, Halle, Germany; 12 Department of Gynecology, Hamburg-Eppendorf University Medical Center, Hamburg, Germany; 13 ClinSol GmbH & Co KG, Würzburg, Germany; 14 Department of Gynecology and Obstetrics, Technische Universität Dresden Germany and National Center for Tumor Diseases (NCT/UCC), Dresden, Germany; 15 German Cancer Research Center (DKFZ), Heidelberg, Germany; 16 Faculty of Medicine and University Hospital Carl Gustav Carus, Technische Universität Dresden, Dresden, Germany; 17 Helmholtz-Zentrum Dresden - Rossendorf (HZDR), Dresden, Germany; 18 Department of Gynecology and Obstetrics, Helios Clinics Berlin-Buch, Berlin, Germany; 19 Institute of Human Genetics, University Hospital Erlangen, Comprehensive Cancer Center Erlangen-EMN, Erlangen, Germany; 20 Department of Gynecology and Obstetrics, Marienhospital Bottrop, Bottrop, Germany; 21 Institute of Pathology, University Hospital Erlangen, Comprehensive Cancer Center Erlangen-EMN, Erlangen, Germany; 22 Department of Gynecology and Obstetrics, University Hospital Freiburg, Freiburg, Germany; 23 Department of Gynecology and Obstetrics, University Hospital Düsseldorf, Düsseldorf, Germany; 24 Center for Integrated Oncology Aachen Bonn Köln Düsseldorf, Düsseldorf, Germany; 25 Division of Gynecologic Oncology, National Center for Tumor Diseases, University Hospital and German Cancer Research Center, Heidelberg, Germany; 26 Biostatistics Unit, Erlangen University Hospital, Department of Gynecology and Obstetrics, Comprehensive Cancer Center Erlangen-EMN, Friedrich-Alexander Universität Erlangen-Nürnberg, Erlangen, Germany

**Keywords:** Breast cancer, Cancer genetics

## Abstract

Germline mutations in *BRCA1* and *BRCA2* (g*BRCA1/2*) are required for a PARP inhibitor therapy in patients with HER2-negative (HER2−) advanced breast cancer (aBC). However, little is known about the prognostic impact of *gBRCA1/2* mutations in aBC patients treated with chemotherapy. This study aimed to investigate the frequencies and prognosis of germline and somatic *BRCA1/2* mutations in HER2- aBC patients receiving the first chemotherapy in the advanced setting. Patients receiving their first chemotherapy for HER2- aBC were retrospectively selected from the prospective PRAEGNANT registry (NCT02338167). Genotyping of 26 cancer predisposition genes was performed with germline DNA of 471 patients and somatic tumor DNA of 94 patients. Mutation frequencies, progression-free and overall survival (PFS, OS) according to germline mutation status were assessed. g*BRCA1/2* mutations were present in 23 patients (4.9%), and 33 patients (7.0%) had mutations in other cancer risk genes. Patients with a g*BRCA1/2* mutation had a better OS compared to non-mutation carriers (HR: 0.38; 95%CI: 0.17–0.86). PFS comparison was not statistically significant. Mutations in other risk genes did not affect prognosis. Two somatic *BRCA2* mutations were found in 94 patients without *gBRCA1/2* mutations. Most frequently somatic mutated genes were *TP53* (44.7%), *CDH1* (10.6%) and *PTEN* (6.4%). In conclusion, aBC patients with g*BRCA1/2* mutations had a more favorable prognosis under chemotherapy compared to non-mutation carriers. The mutation frequency of ~5% with g*BRCA1/2* mutations together with improved outcome indicates that germline genotyping of all metastatic patients for whom a PARP inhibitor therapy is indicated should be considered.

## Introduction

Effective therapy options have been recently developed for patients with advanced breast cancer (aBC)^1^. For HER2-negative, hormone receptor-positive tumors, three different hormone therapy-based options have received approval: everolimus, cyclin-dependent kinase 4 and 6 (CDK4/6)-inhibitors and alpelisib. For triple-negative breast cancer (TNBC) patients, the checkpoint inhibitors atezolizumab and pembrolizumab as well as the antibody-drug conjugate sacituzumab govitecan^1^ were approved in their respective indications. The poly (ADP-ribose) polymerase (PARP)-inhibitors (PARPi) olaparib and talazoparib were approved for all HER2-negative subtypes^2,3^, in case of a germline *BRCA1* or *BRCA2* mutation (g*BRCA1/2*m).

The PARPi registration studies, which have been conducted in aBC patients with a g*BRCA1/2*m, were both randomized studies which compared the PARPi to a chemotherapy at physician’s choice^2,3^. For the OlympiAD study (olaparib) these were capecitabine, eribulin and vinorelbine. In the EMBRACA study (talazoparib) gemcitabine was also allowed. Both studies showed a superior progression-free survival (PFS) in favor of the PARPi^2,3^.

However, not much is known about the effect of g*BRCA1/2*m on the prognosis of patients with aBC and their possible influence on the therapy response. For patients with aBC, the TNT trial of advanced TNBC patients randomized to carboplatin or docetaxel provided insight into therapeutic interactions^4^. Among treatment with docetaxel, there was no difference between patients with or without a g*BRCA1/2*m. However, in patients treated with carboplatin, patients with a g*BRCA1/2*m had a more favorable prognosis than patients with a wildtype genotype^4^. Separately, several studies in the neoadjuvant setting have shown a higher pathological complete response rate (pCR) for patients with a g*BRCA1/2*m^5–7^. In addition, there is little data available on the efficacy of directed therapies among women with mutations in other BC risk genes. One small study has suggested that aBC patients with a *PALB2* mutation may benefit from therapy with olaparib^8^.

The aim of this study was to assess the effect of g*BRCA1/2*m on the prognosis (PFS and overall survival (OS)) of HER2-negative aBC patients treated with the first chemotherapy in the advanced setting. Furthermore, the frequencies and the prognostic effect of germline and somatic mutations in BC risk panel genes were analyzed.

## Results

### Patient and tumor characteristics

Patients were retrospectively selected for genetic testing form the prospective PRAEGNANT registry (NCT02338167^9^). The patient flow chart is presented in Supplementary Fig. 1. Among the 471 patients of the main population (germline genotyping data and prognostic information available), a total of 23 (4.9%) *gBRCA1/2*m and 33 (7.0%) germline mutations in the remaining 24 BC risk genes were identified. Patient characteristics are shown in Table 1. Patients with a *BRCA1/2*m were on average 51.0 years old, while patients without a *BRCA1/2*m or a mutation in another BC risk gene were on average 58.8 and 60.2 years old. Patients with a g*BRCA1/2*m more frequently had TNBC (*N* = 6; 26.1%) than patients without a *BRCA1/2*m (15.9%) or patients with a mutation in one of the other BC risk genes (12.1%). Also, patients with a *BRCA1/2*m had higher grade tumors with 56.5% having a grading of 3 (Table 1). Treatment characteristics are shown in Table 2. Patients with a g*BRCA1/2*m were more frequently treated with a platinum-based chemotherapy (*N* = 8; 34.8%) than non-*BRCA1/2*m patients (*N* = 47; 10.5%), although platinum-based chemotherapy was also dependent on hormone receptor status and more frequently given to hormone receptor-negative (i.e., TNBC) than hormone receptor-positive patients (Table 2). No greater differences in other treatment characteristics were observed. Furthermore, 55.6% of hormone receptor-positive patients received at least one line of endocrine therapy before starting chemotherapy for aBC, while 89.5% of hormone receptor-negative (i.e., TNBC) patients received first-line chemotherapy (Supplementary Table 1). Common patient and tumor characteristics and associations between mutation status groups and common genotyping criteria are shown in Supplementary Table 2.

**Table 1:** Patient disease characteristics according to germline mutation status (*N* = 471)

Characteristics		No mutation (*N* = 415)	g*BRCA1/2*m (*N* = 23)	Other BC risk genes (*N* = 33)
Age at study entry (years)	*N* with value	415	23	33
	Mean (SD)	58.8 (12.6)	51.0 (9.7)	60.2 (12.9)
	Missing values	0	0	0
BMI (kg/m^2^)	N with value	370	20	32
	Mean (SD)	25.5 (4.7)	25.9 (5.1)	27.6 (5.4)
	Missing values	45 (10.8)	3 (13.0)	1 (3.0)
Hormone receptor status	HR-	66 (15.9)	6 (26.1)	4 (12.1)
	HR+	343 (82.7)	17 (73.9)	28 (84.8)
	Missing values	6 (1.4)	0 (0.0)	1 (3.0)
Tumor grade	1	21 (5.1)	1 (4.3)	0 (0.0)
	2	231 (55.7)	9 (39.1)	15 (45.5)
	3	133 (32.0)	13 (56.5)	14 (42.4)
	Missing values	30 (7.2)	0 (0.0)	4 (12.1)
Therapy line	1	209 (50.4)	16 (69.6)	19 (57.6)
	2	82 (19.8)	3 (13.0)	4 (12.1)
	3	75 (18.1)	3 (13.0)	6 (18.2)
	4+	49 (11.8)	1 (4.3)	4 (12.1)
	Missing values	0 (0.0)	0 (0.0)	0 (0.0)
ECOG performance status	0	188 (45.3)	11 (47.8)	17 (51.5)
	1	158 (38.1)	8 (34.8)	15 (45.5)
	2+	41 (9.9)	2 (8.7)	1 (3.0)
	Missing values	28 (6.7)	2 (8.7)	0 (0.0)
Metastasis pattern	Brain	30 (7.2)	1 (4.3)	3 (9.1)
	Visceral	244 (58.8)	15 (65.2)	20 (60.6)
	Bone	47 (11.3)	3 (13.0)	5 (15.2)
	Others	88 (21.2)	3 (13.0)	5 (15.2)
	Missing values	6 (1.4)	1 (4.3)	0 (0.0)
Concomitant diseases	0 or 1	198 (47.7)	15 (65.2)	14 (42.4)
	2 to 4	141 (34.0)	5 (21.7)	17 (51.5)
	5+	53 (12.8)	1 (4.3)	2 (6.1)
	Missing values	23 (5.5)	2 (8.7)	0 (0.0)

*BMI* body mass index, *SD* standard deviation, *HR* hormone receptor

**Table 2:** Chemotherapies at study entry independent from therapy lines according to genomic *BRCA* (g*BRCA*) mutation and hormone receptor (HR) status (*N* = 471)

Chemotherapy	g*BRCA* mutation status		Hormone receptor status	
	Mutation (*N* = 23)	Wildtype (*N* = 448)	Positive (*N* = 388)	Negative (*N* = 76)
Anthracyline taxane combination	2 (8.7)	32 (7.1)	32 (8.2)	1 (1.3)
Anthracycline monotherapy	1 (4.3)	56 (12.5)	56 (14.4)	0 (0.0)
Taxane monotherapy	6 (26.1)	100 (22.3)	88 (22.7)	16 (21.1)
Bevacizumab treatments	4 (17.4)	106 (23.7)	89 (22.9)	20 (26.3)
Platinum-based therapy	8 (34.8)	47 (10.5)	30 (7.7)	24 (31.6)
Eribulin	1 (4.3)	31 (6.9)	20 (5.2)	11 (14.5)
Capecitabine monotherapy	1 (4.3)	45 (10.0)	43 (11.1)	3 (3.9)
Capecitabine taxane combination	0 (0.0)	7 (1.6)	7 (1.8)	0 (0.0)
Vinorelbine monotherapy	0 (0.0)	11 (2.5)	11 (2.8)	0 (0.0)
Other	0 (0.0)	13 (2.9)	12 (3.1)	1 (1.3)

Treatment patterns for number of cycles per line, schedules, durations and reason for discontinuation are not available. Data is presented as *N* (%)

### Detailed genotyping results

Germline genotyping results for the 26 genes of interest are shown in Table 3. *BRCA1*m were found in 10 (2.1%) and *BRCA2*m in 13 (2.8%) of the 471 aBC patients. *CHEK2* germline mutations were found in 9 patients (1.9%), and *PALB2* mutations in 7 patients (1.5%). A list of all mutations found is shown in Supplementary Table 3.

**Table 3:** Mutation genotyping results on patient level (population for germline genotyping *N* = 471; population for somatic genotyping *N* = 94)

Gene name	Germline mutation (*N* = 471)	Somatic mutation (*N* = 94)
*APC*	1 (0.2)	0 (0.0)
*ATM*	1 (0.2)	4 (4.3)
*BARD1*	1 (0.2)	0 (0.0)
*BRCA1*	10 (2.1)	0 (0.0)
*BRCA2*	13 (2.8)	2 (2.1)
*BRIP1*	1 (0.2)	0 (0.0)
*CDH1*	1 (0.2)	10 (10.6)
*CDKN2A*	0 (0.0)	1 (1.1)
*CHEK2*	9 (1.9)	4 (4.3)
*FANCC*	2 (0.4)	0 (0.0)
*KRAS*	0 (0.0)	2 (2.1)
*MEN1*	0 (0.0)	3 (3.2)
*MRE11A*	2 (0.4)	1 (1.1)
*MSH6*	2 (0.4)	0 (0.0)
*MUTYH*	1 (0.2)	1 (1.1)
*NF1*	0 (0.0)	5 (5.3)
*PALB2*	7 (1.5)	1 (1.1)
*PMS2*	1 (0.2)	0 (0.0)
*PTEN*	1 (0.2)	6 (6.4)
*RAD51C*	2 (0.4)	0 (0.0)
*RAD51D*	1 (0.2)	1 (1.1)
*TP53*	1 (0.2)	42 (44.7)
*XRCC2*	1 (0.2)	0 (0.0)

Somatic genotyping results were available from 94 tumors from patients negative for g*BRCA1/2*m (Table 3). Two (2.1%) further *BRCA2*m were found within this population. Most frequent tumor mutations were in *TP53* (*N* = 42; 44.7%), *CDH1* (*N* = 10; 10.6%), *PTEN* (*N* = 6; 6.4%) and *NF1* (*N* = 5; 5.3%). Patient characteristics according to somatic mutation status are shown in Supplementary Table 4. An overview of all mutations, copy number variations and rearrangements is shown in Supplementary Tables 5–8.

### Influence of germline mutations on prognosis in aBC patients treated with chemotherapy

**Fig. 1:**
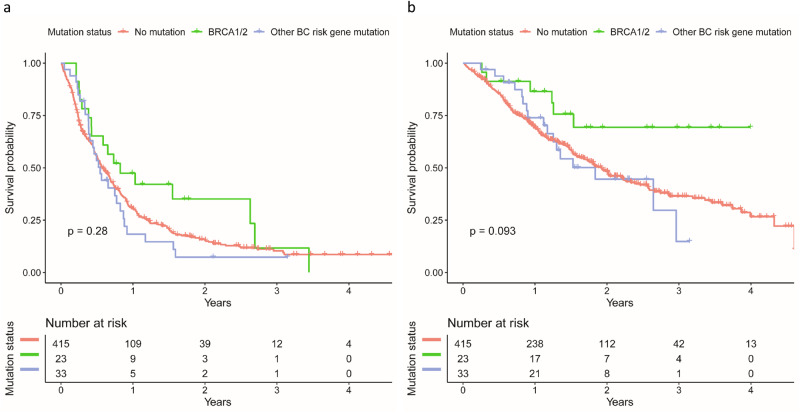
Effect of germline mutation status on survival. **a** Effect on progression-free survival and (**b**) overall survival. Red graph represents the patient population without any germline mutations. Green graph are patients with a germline *BRCA1* or *BRCA2* mutation. Blue graph depicts patients with mutations in other known breast cancer (BC) risk genes.

The influence of g*BRCA1/2* and other BC risk gene mutations on PFS and OS is shown in Table 4. Median follow-up time was 6.5 months for PFS and 14.9 months for OS. g*BRCA1/2*m had a statistically significant effect on OS (hazard ratio (HR): 0.38; 95%CI: 0.17–0.86; *P* = 0.02), whereas the effect on PFS did not reach statistical significance (HR: 0.68; 95%CI: 0.42–1.12; *P* = 0.13). The respective Kaplan Meier curves with log-rank *P* values are shown in Fig. 1a and b. An influence of mutations in genes other than g*BRCA1/2* on PFS (HR: 1.15; 95%CI: 0.78–1.71; *P* = 0.48) or OS (HR: 1.11; 95%CI: 0.67–1.83; *P* = 0.70) could not be shown. Median PFS was 6.9 months (95%CI: 6.1–8.2) in patients without a mutation, 9.9 months (95%CI: 5.1-not reached) in patients with a g*BRCA1/2*m, and 6.5 months (95%CI: 4.8–10.4) in patients with a mutation in one of the remaining BC risk genes. With regard to OS, median survival time was not reached by patients with g*BRCA1/2*m, was 23.1 months (95%CI; 19.5–27.2) for patients without a mutation and 22.0 months (95%CI: 15.0–not reached) in patients with a mutation in the other BC risk genes.

**Table 4:** Unadjusted and adjusted hazard ratios (PFS and OS) for germline mutation status and somatic mutation status

Outcome	Type	Mutation	Unadjusted analysis		Adjusted analysis^a^	
			HR^b^ (95% CI)^b^	*P* value	HR^a^ (95% CI)^b^	*P* value
PFS	Germline	g*BRCA*m	0.70 (0.43, 1.13)	0.15	0.68 (0.42, 1.12)	0.13
		Any other mutation	1.11 (0.76, 1.64)	0.59	1.15 (0.78, 1.71)	0.48
OS	Germline	g*BRCA*m	0.42 (0.19, 0.94)	0.04	0.38 (0.17, 0.86)	0.02
		Any other mutation	1.04 (0.63, 1.70)	0.89	1.11 (0.67, 1.83)	0.70

*HR* hazard ratio, *CI* confidence interval, *PFS* progression-free survival, *OS* overall survival

^a^HRs are adjusted for age at study entry, hormone receptor status, tumor grade, therapy line, ECOG performance status, metastasis pattern, and number of concomitant diseases

^b^Reference category is “no mutation”

In an exploratory approach we generated Kaplan Meier curves according to specific functionally defined groups of genes (*BRCA1/2* vs. *PALB2* vs. *CHEK2* vs. other homologous recombination (HRR) genes vs. other DNA repair genes vs. the remaining BC risk genes) (Supplementary Table 9). These are shown in Supplementary Figs. 2 and 3.

## Discussion

In this analysis of HER2-negative aBC patients treated with the first chemotherapy in the advanced setting, we evaluated frequencies of germline mutations in BC risk genes and showed that 4.9% of patients had a *BRCA1/2*m and about 1.5% of patients had a *PALB2* mutation. In a subset analysis of 94 tumors of non-g*BRCA1/2* patients, 2 further likely somatic *BRCA2* mutations (2.1%) were identified. Patients with a g*BRCA1/2*m undergoing the first chemotherapy for aBC had a better prognosis.

This study may allow additional interpretation of the results from the comparator arms of the large phase III studies that compared the PARPis olaparib and talazoparib with chemotherapy of physician’s choice^2,3^. Previous therapy with platinum chemotherapies was allowed in the (neo)adjuvant setting if a recurrence had not occurred within 6 months (EMBRACA) or 12 months (OlympiAD) and if a previous therapy in the metastatic setting did not result in disease progression. Subgroup analyses for patients that did not receive prior chemotherapy for aBC in the OlympiAD trial showed a median PFS of 8.1 months (95%CI: 5.6–8.5) with olaparib vs. 4.1 months (95%CI: 2.8–7.7) with chemotherapy^10^. In the EMBRACA study, median PFS with chemotherapy was 8.7 months (95%CI: 5.5–18.0) and 9.9 months (95%CI: 8.5–13.3) with talazoparib^11^. In the current study, median PFS of patients with *BRCA1/2* mutations was comparable to that of EMBRACA and OlympiAD with 9.9 months (95%CI: 5.1–not reached) and was located between the results of the two trails in the group of patients without a *BRCA1/2*m (6.9 months; 95%CI: 6.1–8.2). Patients in the chemotherapy arm of OlympiAD had a median OS of 14.7 months and patients in the olaparib arm of 22.6 months^12^. The median OS in our study was 23.1 months for patients without a germline mutation and the median OS was not reached in the group of patients with a g*BRCA1/2*m. As our patient population contained a higher percentage of patients with hormone receptor-positive BC than the population of the OlympiAD trial, this could reflect the observed difference in OS.

It has to be considered that in the current study a different pattern of chemotherapy was used. While most patients in the OlympiAD study were treated with capecitabine (45%), eribulin (37%) and vinorelbine (18%), in the current study only 21% of the patients without and 8.6% with a *BRCA1/2*m were treated with the physician’s choice chemotherapy options from the OlympiAD study. Additionally, consistent with routine clinical practice, platinum-based chemotherapies were widely used in the *BRCA1/2*-positive population (34.8%) and in 10.5% of patients without a *BRCA1/2*m and there was wide use of taxane-based treatments. Nevertheless, it has to be noted that the choice of chemotherapy was also dependent on hormone receptor status. Regardless of these major differences from the PARPi studies, our comparison of mutation carriers vs. non-carriers showed a significantly better OS and better PFS for patients with *BRCA1/2*m, indicating that in patients with g*BRCA1/2*m, chemotherapy may have better efficacy. Similarly, a better efficacy, in response to neoadjuvant therapy has been observed for *BRCA1/2*m carriers^5–7,13^.

Mutations in other BC risk genes did not seem to have a larger influence on the prognosis in this population that was treated with chemotherapy, perhaps due to gene-specific effects on therapeutic response. However, this population may be a preferred target population for PARPi treatments. For example, therapy with olaparib is specifically effective in patients with a *PALB2* mutation^8^. In addition, 6.4% of tumors had a mutation in *PTEN*, which is possibly relevant for current studies with *PIK3CA* inhibitors^14^.

Another aim was to determine the tumor mutation rate in patients with a negative g*BRCA1/2*m status. There are few data available describing this mutation frequency. We found a s*BRCA2*m in 2 out of 94 patients and no *BRCA1*m. This frequency of 2.1% should be interpreted with caution because of the small number. There is evidence that somatic mutations are present in 6.3% of all ovarian cancer patients^15^. However, this has to be seen in relation to the 20.5% of the ovarian cancer patients with germline *BRCA1/2* mutations. In comparison, we observed 4.9% (*N* = 23) with germline and 2.1% (*N* = 2) with somatic *BRCA1/2*m. PARPi olaparib and talazoparib are only approved for treatment of metastatic BC patients based on g*BRCA1/2*m and no clinical evidence has been generated indicating a benefit for selected therapies based on somatic mutation status of *BRCA1/2*. A study focusing on that issue would have to include larger screening efforts to identify patients with a positive somatic mutation in *BRCA1/2* but negative germline mutation status.

There are limitations to this analysis. First, it is a retrospective analysis with a prospective data collection. The genotyping was not performed according to any time-dependent patient characteristics. However, blood samples were drawn at baseline to exclude a follow-up bias. Results from our retrospective analyses did therefore not directly affect subsequent treatment. However, it has to be noted that from the 23 patients with a g*BRCA*1/2 mutation in our dataset, 13 had previously been tested for g*BRCA* mutations as part of clinical routine care. It remains unclear whether, and to which extent, these results influenced the patient´s treatment. Unfortunately, we were also unable to provide detailed information on subsequent therapy lines. As the evaluation of the impact of subsequent therapies on OS is of interest, this should be a focus of future research. Furthermore, tumor sample genotyping was carried out for a small subset. However, patient characteristics for the complete study population and the subset for somatic genotyping were very similar.

With regard to prognosis, patients with g*BRCA1/2* mutations were more frequently treated with platinum-based chemotherapy regimens, which could add to a better outcome in this population.

In conclusion it can be hypothesized, that HER2-negative aBC patients with a g*BRCA1/2*m have a greater benefit from first-line chemotherapy than non-carriers or those with mutations in other BC risk genes. Furthermore, the efficacy of certain chemotherapies or PARPi for treatment of the approximately 2% of metastatic BC patients with s*BRCA1/2*m should be considered.

## Methods

### Patients

Patients with advanced or metastatic disease were eligible for inclusion into the prospective PRAEGNANT registry (NCT02338167^9^, ongoing) at any timepoint during the course of their disease. Research was conducted in accordance to the Declaration of Helsinki. All patients provided written informed consent and the study was approved by the ethics committees (ethical approval number: 234/2014BO1: first approval on June 17 2014, approval of Amendment 1 on June 11 2015, approval of Amendment 2 on March 18 2019; Ethics Committee of the Medical Faculty, University of Tübingen, Tübingen, Germany). Blood samples were collected at inclusion into the registry. Genetic testing was performed as part of the scientific evaluation of all patients included into the PRAEGNANT study (2728 patients registered in PRAEGNANT between 07/2014 and 09/2018 at 47 study sites). Germline and somatic testing were done retrospectively. For survival analysis, patients were excluded in the following hierarchical order: 440 HER2-positive patients, 201 patients with incomplete documentation, 768 patients that did not receive chemotherapy, 723 patients not prospectively included (>90 days after therapy start), 1 patient under PARPi therapy, 66 patients without gBRCA1/2 results and 58 patients with insufficient follow-up data. The remaining 471 patients with germline genotyping data and prognostic information were included in this analysis. Somatic testing was performed for patients without a gBRCA1/2 mutation from whom tumor tissue was available. A patient flow chart is shown in Supplementary Fig. 1. Clinical data collection^9^ and definition of hormone receptor, HER2 status and grading are described in the Supplementary Methods. Data categories captured are described in Supplementary Table 10.

### Germline genotyping

Germline DNA was extracted from whole blood using an automated chemagic MSM-I-system (Perkin-Elmer, Baesweiler, Germany). DNA concentration was measured by the QuantiFluor®dsDNA System (Promega, Mannheim, Germany). Mutation testing of 746 target regions covering all coding regions and consensus splice sites from 37 cancer predisposition genes was performed using a custom amplicon-based QIAseq panel (QIAGEN, Hilden, Germany) and sequencing as previously described^16^. Libraries were individually bar-coded by dual indexing and subjected to paired-end 150 bp sequencing in pools of 768 per lane of a HiSeq4000. The median sequence read depth per nucleotide was 200X with 99.7% of target regions yielding >20X reads in all samples. Sequence realignment, recalibration, haplotype calling, and depth of coverage were conducted using Genome Analysis Toolkit (GATK) version 3.4-46^17^. Copy number variation (CNV) was detected with Pattern CNV v1.1.3^18^. Annotation of mutations was conducted using American College of Medical Genetics and Association for Molecular Pathology guidelines^19^. Missense mutations were annotated as pathogenic and likely pathogenic according to ClinVar^20^. Low penetrance missense variants in CHEK2 were excluded from analyses. The 37 gene QIAseq assay was previously validated as having > 99% analytical sensitivity and specificity for single nucleotide variants, insertions and deletions <15 bp in length, and exon-level deletions and duplications^21^. For this analysis, 26 cancer predisposition genes, that were also available on the somatic genotyping panel (Foundation Medicine, Inc., Cambridge, MA, USA), were considered (see below). A list of all gene classifications is shown in Supplementary Tables 9 and 11.

### Somatic genotyping

After germline genotyping, tumor material that was available from patients without a g*BRCA1/2*m was subjected to somatic sequencing (Foundation Medicine, Inc.). Out of 139 available tumor samples, sequencing data from 111 passed quality control and 17 were subsequently excluded due to missing follow-up information, resulting in 94 patients with data on somatic mutation status for survival analysis.

### Statistical analysis

Continuous characteristics are presented as means and standard deviations (SD). Categorical characteristics are presented as frequencies and percentages.

PFS was defined as the time from the date of initiation of therapy to the earliest date of disease progression (distant metastasis, local recurrence, death from any cause) or the last known progression-free date. Observation time was left-truncated for the time at which the patient entered the study if study entry was later than the start of treatment. OS was defined in a similar fashion.

The primary objective was to investigate whether the mutation status influenced survival in addition to well-known prognostic patient and tumor characteristics. A multivariable Cox regression model (basic model) was fitted with PFS as outcome and the following predictors: age at study entry, hormone receptor status (positive/negative), HER2 status (positive/negative), tumor grade, selected therapy line, ECOG status, metastasis pattern, and number of concomitant diseases. Subsequently, a Cox model was fitted containing the mutation status (no mutation, *BRCA1/2*m, other mutation) and the predictors of the basic model. Both models were compared using a likelihood ratio test (LRT). A significant *P* value would indicate that gene mutations influenced survival additionally to the considered prognostic factors. Adjusted hazard ratios (HRs) for mutation status were calculated using the extended Cox model.

Similar analyses were performed for OS. As sensitivity analyses, unadjusted HRs were estimated using univariable Cox regression models. Unadjusted survival rates were estimated using the Kaplan–Meier product limit method.

Missing predictor values were imputed as done by Salmen et al. ^22^. The proportional hazards assumptions were checked using the method of Grambsch and Therneau^23^. All of the tests were two-sided, and a *P* value of <0.05 was regarded as statistically significant. Calculations were carried out using the R-system for statistical computing (version 3.6.1; R Development Core Team, Vienna, Austria, 2017).

### Supplementary information

Supplementary files

## Data Availability

Data used for this article cannot be shared in full due to the nature of the data (many mutations only occurred in one individual) and possible identification of the human participants.

## Code availability

No custom computer codes or algorithms were used in the current study.
