# Supplementary material for: Susceptibility gene mutations in germline and tumors of patients with HER2-negative advanced breast cancer
Source: NPJ Breast Cancer. 2024 Jul 13;10:57. doi: 10.1038/s41523-024-00667-x (PMC11246424; doi:10.1038/s41523-024-00667-x)
Supplement: Supplementary file 1 — Supplementary files [file 41523_2024_667_MOESM1_ESM.pdf]

Supplementary files

Susceptibility gene mutations in germline and tumors of patients with HER2-negative advanced breast cancer

*Peter A. Fasching [1], Chunling Hu [2], Steven N. Hart [3], Matthias Ruebner [1], Eric C. Polley [3], Rohan D. Gnanaolivu [3], Andreas D. Hartkopf [4], Hanna Huebner [1], Wolfgang Janni [5], Peyman Hadji [6], Hans Tesch [7], Sabrina Uhrig [1], Johannes Ettl [8], Michael P. Lux [9], Diana Lüftner [10], Markus Wallwiener [11], Lena A. Wurmthaler [1], Chloë Goossens [1], Volkmar Müller [12], Matthias W. Beckmann [1], Alexander Hein[1], Daniel Anetsberger [1], Erik Belleville [13], Pauline Wimberger [14,15,16,17], Michael Untch [18], Arif B. Ekici [19], Hans-Christian Kolberg [20], Arndt Hartmann [21], Florin-Andrei Taran [22], Tanja N. Fehm [23,24], Diethelm Wallwiener [4], Sara Y. Brucker [4], Andreas Schneeweiss [25], *Lothar Häberle [1,26], *Fergus J. Couch [2,3]

These authors contributed equally to this work

[1] Department of Gynecology and Obstetrics, Erlangen University Hospital, Comprehensive Cancer Center Erlangen-EMN, Friedrich-Alexander Universität Erlangen-Nürnberg, Erlangen, Germany
[2] Department of Laboratory Medicine and Pathology, Mayo Clinic, Rochester, MN, USA
[3] Department of Quantitative Health Sciences, Mayo Clinic, Rochester, MN, USA
[4] Department of Obstetrics and Gynecology, University of Tübingen, Tübingen, Germany
[5] Department of Gynecology and Obstetrics, Ulm University Hospital, Ulm, Germany
[6] Frankfurt Center for Bone Health, Frankfurt am Main, Germany
[7] Oncology Practice, Bethanien Hospital, Frankfurt am Main, Germany
[8] Department of Obstetrics and Gynecology, Klinikum rechts der Isar, Technical University of Munich, Munich, Germany
[9] Department of Gynecology and Obstetrics, Frauenklinik St. Louise, Paderborn, St. Josefs-Krankenhaus, Salzkotten, Germany; St. Vincenz Kliniken Salzkotten + Paderborn, Paderborn, Germany
[10] Immanuel Klinik Märkische Schweiz & Medical University of Brandenburg Theodor Fontane, Rüdersdorf bei Berlin, Germany
[11] Department of Gynecology, Halle University Hospital, Halle, Germany
[12] Department of Gynecology, Hamburg-Eppendorf University Medical Center, Hamburg, Germany
[13] ClinSol GmbH & Co KG, Würzburg, Germany
[14] Department of Gynecology and Obstetrics, Technische Universität Dresden Germany and National Center for Tumor Diseases (NCT/UCC), Dresden, Germany
[15] German Cancer Research Center (DKFZ), Heidelberg, Germany
[16] Faculty of Medicine and University Hospital Carl Gustav Carus, Technische Universität Dresden, Dresden, Germany
[17] Helmholtz-Zentrum Dresden - Rossendorf (HZDR), Dresden, Germany
[18] Department of Gynecology and Obstetrics, Helios Clinics Berlin-Buch, Berlin, Germany
[19] Institute of Human Genetics, University Hospital Erlangen, Comprehensive Cancer Center Erlangen-EMN, Erlangen, Germany
[20] Department of Gynecology and Obstetrics, Marienhospital Bottrop, Bottrop, Germany
[21] Institute of Pathology, University Hospital Erlangen, Comprehensive Cancer Center Erlangen-EMN, Erlangen, Germany
[22] Department of Gynecology and Obstetrics, University Hospital Freiburg, Germany
[23] Department of Gynecology and Obstetrics, University Hospital Düsseldorf, Germany
[24] Center for integrated oncology Aachen Bonn Köln Düsseldorf, Düsseldorf, Germany
[25] Division of Gynecologic Oncology, National Center for Tumor Diseases, University Hospital and German Cancer Research Center, Heidelberg, Germany
[26] Biostatistics Unit, Erlangen University Hospital, Department of Gynecology and Obstetrics, Comprehensive Cancer Center Erlangen-EMN, Friedrich-Alexander University Erlangen-Nuremberg, Germany

Supplementary Methods

Clinical Data collection

Data were collected and documented in electronic case report forms by trained staff [1] and monitored using automated plausibility checks and on-site monitoring. Epidemiological data such as family history, cancer risk factors, quality of life, nutrition and lifestyle items and psychological health that are not usually documented as part of routine clinical work, were collected prospectively using structured paper questionnaires (Supplementary Table S10).

Definition of hormone receptor, HER2 status, and grading

Definition of tumor hormone receptor status, HER2 status and grade was described before [2]. Briefly, estrogen receptor (ER) status, progesterone receptor (PR) status, HER2 status and grade were requested for biopsies from the right breast, left breast, local recurrence, or metastatic site of each tumor. If a biomarker assessment of the metastatic site was available, the receptor status was used for this analysis. In case of missing information from metastases, the latest biomarker results from the primary tumor were used. Additionally, all patients ever treated with prior endocrine therapy were assumed to be hormone receptor positive and all patients ever treated with an anti-HER2 therapy were assumed to be HER2 positive. There was no central review of biomarkers. ER and PR were considered positive if ≥1% of tumor cells were stained. A positive HER2 status required an IHC score of 3+ or a positive FISH/CISH.

Supplementary references

1. Fasching PA, Brucker SY, Fehm TN et al. Biomarkers in Patients with Metastatic Breast Cancer and the PRAEGNANT Study Network. Geburtshilfe Frauenheilkd 2015; 75: 41-50.

2. Hartkopf AD, Huober J, Volz B et al. Treatment landscape of advanced breast cancer patients with hormone receptor positive HER2 negative tumors - Data from the German PRAEGNANT breast cancer registry. Breast 2018; 37: 42-51.

Supplementary Tables

Supplementary Table 1: Therapy line in which chemotherapy was started, according to hormone receptor status. Data is presented as *N* (%)

**Therapy line**	**Hormone receptor status**	
	**Positive (*N*=388)**	**Negative (*N*=76)**
1	172 (44.3)	68 (89.5)
2	80 (20.6)	7 (9.2)
3	83 (21.4)	1 (1.3)
4+	53 (13.7)	0 (0.0)

Supplementary Table 2: Breast Cancer Risk Factors in correlation to germline genotyping results. Data is presented as *N* (%) [BC: breast cancer; TNBC: triple-negative breast cancer]

**Characteristics**		**No mutation (*N*=415)**	**gBRCAm (*N*=23)**	**Other BC Risk Genes (*N*=33)**
Diagnosis before age 46	No	298 (71.8)	12 (52.2)	24 (72.7)
	Yes	117 (28.2)	11 (47.8)	9 (27.3)
	Missing values	0 (0.0)	0 (0.0)	0 (0.0)
Bilateral BC before age 51	No	402 (96.9)	17 (73.9)	31 (93.9)
	Yes	13 (3.1)	6 (26.1)	2 (6.1)
	Missing values	0 (0.0)	0 (0.0)	0 (0.0)
Diagnosis before age 51 and one further 1st line relative with BC	No	393 (94.7)	18 (78.3)	30 (90.9)
	Yes	22 (5.3)	5 (21.7)	3 (9.1)
	Missing values	0 (0.0)	0 (0.0)	0 (0.0)
Diagnosis before age 61 and TNBC	No	372 (89.6)	17 (73.9)	30 (90.9)
	Yes	43 (10.4)	6 (26.1)	3 (9.1)
	Missing values	0 (0.0)	0 (0.0)	0 (0.0)
Diagnosis after age 50 and one 1st line relative with BC under age 51	No	412 (99.3)	22 (95.7)	33 (100.0)
	Yes	3 (0.7)	1 (4.3)	0 (0.0)
	Missing values	0 (0.0)	0 (0.0)	0 (0.0)
More than 2 BC cases in this family	No	409 (98.6)	22 (95.7)	31 (93.9)
	Yes	6 (1.4)	1 (4.3)	2 (6.1)
	Missing values	0 (0.0)	0 (0.0)	0 (0.0)
1st line relative with ovarian cancer	No	404 (97.3)	21 (91.3)	32 (97.0)
	Yes	11 (2.7)	2 (8.7)	1 (3.0)
	Missing values	0 (0.0)	0 (0.0)	0 (0.0)
Male relative with BC	No	405 (97.6)	22 (95.7)	32 (97.0)
	Yes	10 (2.4)	1 (4.3)	1 (3.0)
	Missing values	0 (0.0)	0 (0.0)	0 (0.0)
Own history of ovarian cancer	No	414 (99.8)	22 (95.7)	32 (97.0)
	Yes	1 (0.2)	1 (4.3)	1 (3.0)
	Missing values	0 (0.0)	0 (0.0)	0 (0.0)
Any reason for genotyping	No	250 (60.2)	6 (26.1)	18 (54.5)
	Yes	165 (39.8)	17 (73.9)	15 (45.5)
	Missing values	0 (0.0)	0 (0.0)	0 (0.0)

Supplementary Table 3: List of all mutations and their frequency from the germline genotyping (table on mutation level, not gene level, not patient level) (*N*=471)

Gene Name	HGVS mutation nomenclature	Mutation count in the total population of 471 patients
APC	c.5565T>A_p.Cys1855X	1
ATM	c.3802delG	1
BARD1	c.1872delT	1
BLM	c.1933C>T_p.Gln645X	1
BLM	c.2695C>T_p.Arg899X	1
BRCA1	c.1687C>T_p.Gln563X	1
BRCA1	c.181T>G_p.Cys61Gly	1
BRCA1	c.3481_3491del11	1
BRCA1	c.4065_4068delTCAA	1
BRCA1	c.4932_4933dupAA	1
BRCA1	c.4986+3G>C	1
BRCA1	c.5109T>G_p.Tyr1703X	1
BRCA1	c.5266dupC	3
BRCA2	c.1296_1297delGA	1
BRCA2	c.1813dupA	1
BRCA2	c.2808_2811delACAA	2
BRCA2	c.516+2T>A	1
BRCA2	c.5722_5723delCT	1
BRCA2	c.5857G>T_p.Glu1953X	1
BRCA2	c.7480C>T_p.Arg2494X	1
BRCA2	c.7816_7819dupGACA	1
BRCA2	c.7857G>A_p.Trp2619X	1
BRCA2	c.917dupA	1
BRCA2	c.9196C>T_p.Gln3066X	1
BRIP1	c.3241_3245dup5	1
CDH1	c.377dupC	1
CHEK2	c.1100delC	5
CHEK2	c.1375+1G>A	1
CHEK2	c.444+1G>A	1
CHEK2	exon 9-10 del	2
ERCC2	c.2150C>G_p.Ala717Gly	1
FANCC	c.37C>T_p.Gln13X	1
FANCC	c.455dupA	1
FANCM	c.3603delT	1
FANCM	exon 12-23 del	1
MRE11A	c.1516G>T_p.Glu506X	1
MRE11A	c.1726C>T_p.Arg576X	1
MSH6	c.3226C>T_p.Arg1076Cys	1
MSH6	c.3991C>T_p.Arg1331X	1
MUTYH	c.1171C>T_p.Gln391X	1
PALB2	c.1424dupC	1
PALB2	c.1451T>A_p.Leu484X	1
PALB2	c.2257C>T_p.Arg753X	1
PALB2	c.3456dupA	1

Gene Name	HGVS mutation nomenclature	Mutation count in the total population of 471 patients
PALB2	c.3549C>A_p.Tyr1183X	1
PALB2	c.487_488delGT	1
PALB2	c.509_510delGA	1
PMS2	c.1882C>T_p.Arg628X	1
PPM1D	c.1303dupC	1
PPM1D	c.1451delT	1
PRSS1	c.256C>T_p.Gln86X	1
PRSS1	exon 1-4 del	1
PRSS1	exon 2-5 del	1
PTEN	c.875dupA	1
RAD50	c.3454C>T_p.Arg1152X	1
RAD50	exon 4-14 del	1
RAD51C	c.414G>C_p.Leu138Phe	1
RAD51C	c.706-2A>G	1
RAD51D	c.694C>T_p.Arg232X	1
RECQL	c.1099-2A>C	1
RECQL	c.1476C>G_p.Tyr492X	1
RINT1	c.1333+1G>A	1
TP53	c.734G>T_p.Gly245Val	1
XRCC2	c.96delT	1

Supplementary Table 4: Patient Characteristics according to somatic mutation status (*N*=94)

Characteristics		No mutation (N=33)	sBRCAm (N=2)	Other BC Risk Genes (N=59)
Age at study entry (years)	N with value	33	2	59
	Mean (SD)	61.1 (13.2)	65.5 (10.6)	55.8 (14.0)
	Median (IQR)	64 (50, 72)	65.5 (61.8, 69.2)	56 (46, 66.5)
	Range (min, max)	(35, 82)	(58, 73)	(29, 81)
	Missing values	0	0	0
BMI (kg/m^2)	N with value	30	2	51
	Mean (SD)	25.3 (5.5)	28.2 (0.5)	26.9 (4.7)
	Median (IQR)	25.2 (20.7, 27.8)	28.2 (28.1, 28.4)	26.3 (24.1, 29.9)
	Range (min, max)	(18.4, 41.1)	(27.9, 28.6)	(16.3, 38.9)
	<18.5	1 (3.0)	0 (0.0)	2 (3.4)
	18.5 - <25	13 (39.4)	0 (0.0)	15 (25.4)
	25 - <30	10 (30.3)	2 (100.0)	21 (35.6)
	≥30	6 (18.2)	0 (0.0)	13 (22.0)
	Missing values	3 (9.1)	0 (0.0)	8 (13.6)
Hormone receptor status	HR-	3 (9.1)	0 (0.0)	12 (20.3)
	HR+	30 (90.9)	2 (100.0)	47 (79.7)
	Missing values	0 (0.0)	0 (0.0)	0 (0.0)
Tumor grade	1	0 (0.0)	0 (0.0)	2 (3.4)
	2	21 (63.6)	2 (100.0)	21 (35.6)
	3	10 (30.3)	0 (0.0)	35 (59.3)
	Missing values	2 (6.1)	0 (0.0)	1 (1.7)
Therapy line	1	13 (39.4)	1 (50.0)	34 (57.6)
	2	9 (27.3)	1 (50.0)	15 (25.4)
	3	3 (9.1)	0 (0.0)	7 (11.9)
	4+	8 (24.2)	0 (0.0)	3 (5.1)
	Missing values	0 (0.0)	0 (0.0)	0 (0.0)
ECOG performance status	0	16 (48.5)	2 (100.0)	30 (50.8)
	1	13 (39.4)	0 (0.0)	18 (30.5)
	2+	3 (9.1)	0 (0.0)	7 (11.9)
	Missing values	1 (3.0)	0 (0.0)	4 (6.8)
Metastasis pattern	Brain	1 (3.0)	0 (0.0)	7 (11.9)
	Visceral	21 (63.6)	2 (100.0)	27 (45.8)
	Bone	6 (18.2)	0 (0.0)	7 (11.9)
	Others	5 (15.2)	0 (0.0)	17 (28.8)
	Missing values	0 (0.0)	0 (0.0)	1 (1.7)
Concomitant diseases	0 or 1	16 (48.5)	2 (100.0)	30 (50.8)
	2 to 4	13 (39.4)	0 (0.0)	18 (30.5)
	5+	3 (9.1)	0 (0.0)	8 (13.6)
	Missing values	1 (3.0)	0 (0.0)	3 (5.1)

Supplementary Table 5: Cumulative results of the Foundation Medicine FoundationOne Dx Panel analysis of the somatic genotyping population - Frequencies of patients with a found short variant (SV), copy number alteration (CNA) and rearrangement (REARR)

Gene	Frequency of SV (*N*=94) - *N* (%)	Frequency of CNA (*N*=94) - *N* (%)	Frequency of REARR (*N*=94) - *N* (%)
AKT1	3 (3.2)	1 (1.1)	
AKT3		2 (2.1)	
APC			2 (2.1)
AR		1 (1.1)	
ARAF	1 (1.1)		
ARFRP1		4 (4.3)	
ARID1A	9 (9.6)		
ASXL1	1 (1.1)		1 (1.1)
ATM	4 (4.3)		1 (1.1)
ATR	1 (1.1)		
ATRX	1 (1.1)		
AURKA		3 (3.2)	
AURKB		1 (1.1)	
BCL2L1		2 (2.1)	
BCOR	1 (1.1)		
BRAF			2 (2.1)
BRCA2	2 (2.1)		
BRD4	1 (1.1)		1 (1.1)
BRIP1			2 (2.1)
BTG2			1 (1.1)
CASP8	1 (1.1)		
CBFB	2 (2.1)		
CCND1		23 (24.5)	
CCND2		1 (1.1)	
CCND3		1 (1.1)	
CCNE1		3 (3.2)	
CD274		1 (1.1)	1 (1.1)
CDH1	10 (10.6)		1 (1.1)
CDK6		1 (1.1)	
CDK8		1 (1.1)	
CDKN1B	1 (1.1)		
CDKN2A	1 (1.1)	8 (8.5)	
CDKN2B		6 (6.4)	
CHEK1	1 (1.1)		
CHEK2	4 (4.3)		1 (1.1)
CREBBP	2 (2.1)		
CSF3R		1 (1.1)	
CTNNA1	1 (1.1)	1 (1.1)	
CTNNB1	1 (1.1)		
CUL4A		1 (1.1)	
EMSY		10 (10.6)	
EPHB4		1 (1.1)	
ERBB2	3 (3.2)	4 (4.3)	

**Gene**	**Frequency of SV (*N*=94) - *N* (%)**	**Frequency of CNA (*N*=94) - *N* (%)**	**Frequency of REARR (*N*=94) - *N* (%)**
ERBB3	2 (2.1)		1 (1.1)
ERBB4	1 (1.1)		
ERCC4	1 (1.1)		
ESR1	9 (9.6)	1 (1.1)	
EZH2	1 (1.1)		1 (1.1)
FAM123B			1 (1.1)
FAS		1 (1.1)	
FGF12		1 (1.1)	
FGF19		22 (23.4)	
FGF23		2 (2.1)	
FGF3		21 (22.3)	
FGF4		21 (22.3)	
FGF6		2 (2.1)	
FGFR1		16 (17)	2 (2.1)
FGFR2	1 (1.1)		
FGFR4		2 (2.1)	
FUBP1			1 (1.1)
GABRA6	1 (1.1)		
GATA3	16 (17)		
GNAS		4 (4.3)	1 (1.1)
GRM3	1 (1.1)		
HGF		2 (2.1)	
HRAS		1 (1.1)	
IGF1R		1 (1.1)	
IKBKE		2 (2.1)	
IRS2		2 (2.1)	
JAK2		1 (1.1)	1 (1.1)
JUN		1 (1.1)	
KDM5A		4 (4.3)	
KDM5C	1 (1.1)		1 (1.1)
KDR	1 (1.1)		
KEL	1 (1.1)		
KLHL6	1 (1.1)		
KRAS	2 (2.1)	2 (2.1)	
LYN		6 (6.4)	
MAP2K1	1 (1.1)	1 (1.1)	
MAP2K4	3 (3.2)	7 (7.4)	
MAP3K1	7 (7.4)		
MCL1		4 (4.3)	
MDM2		4 (4.3)	
MDM4		5 (5.3)	
MEN1	3 (3.2)		
MRE11A	1 (1.1)		
MSH3	1 (1.1)		
MTAP		8 (8.5)	
MUTYH	1 (1.1)		
MYB			1 (1.1)

Gene	Frequency of SV (*N*=94) - *N* (%)	Frequency of CNA (*N*=94) - *N* (%)	Frequency of REARR (*N*=94) - *N* (%)
MYC		18 (19.1)	
MYCL1		1 (1.1)	
NBN			1 (1.1)
NF1	6 (6.4)	1 (1.1)	2 (2.1)
NFE2L2	1 (1.1)		
NFKBIA		1 (1.1)	1 (1.1)
NOTCH1	3 (3.2)		
NOTCH2	1 (1.1)		2 (2.1)
NOTCH3		1 (1.1)	
PALB2	1 (1.1)		
PARP1		1 (1.1)	
PDCD1LG2		1 (1.1)	
PIK3C2B		7 (7.4)	1 (1.1)
PIK3CA	30 (31.9)	2 (2.1)	
PIK3R1	5 (5.3)		
POLD1			1 (1.1)
POLE			1 (1.1)
PPP2R1A	1 (1.1)		
PRKCI		1 (1.1)	
PTEN	6 (6.4)	5 (5.3)	2 (2.1)
RAD21		23 (24.5)	
RAD51D	1 (1.1)		
RAF1		1 (1.1)	
RB1	1 (1.1)	1 (1.1)	1 (1.1)
REL	1 (1.1)		
RET	1 (1.1)		
RICTOR		2 (2.1)	
RNF43	1 (1.1)		
ROS1		1 (1.1)	
SDHB	1 (1.1)		
SMAD4	1 (1.1)		
SMARCA4			1 (1.1)
SMARCB1	1 (1.1)		
SOX9	1 (1.1)		
SPEN	7 (7.4)		
SPOP	1 (1.1)		
STK11	1 (1.1)	1 (1.1)	
SUFU		()	1 (1.1)
TBX3	5 (5.3)	1 (1.1)	
TERC		2 (2.1)	
TET2	1 (1.1)		
TNFAIP3	2 (2.1)		
TNFRSF14			1 (1.1)
TP53	42 (44.7)	1 (1.1)	1 (1.1)
VEGFA		2 (2.1)	
VHL	1 (1.1)		
WHSC1L1		17 (18.1)	2 (2.1)

Gene	Frequency of SV (*N*=94) - *N* (%)	Frequency of CNA (*N*=94) - *N* (%)	Frequency of REARR (*N*=94) - *N* (%)
ZNF217		5 (5.3)	1 (1.1)
ZNF703		16 (17)	

Supplementary Table 6: Listing of all mutations and their frequency from the somatic genotyping (list on mutation level, not gene level, not patient level) in *N*=94 patients

Gene	HGVS mutation nomenclature	*N*
AKT1	c.226_227insAGACGGAGCGGCCCCGGCCCAACACCTTCATCATCC; p.I75_R76insQTERPRPNTFII	1
AKT1	c.239_240insCACGGAGCGGCCCCGGCCCAACACCTTCATCATCCGCTGCCTGCAGTG; p.Q79_W80insCTERPRPNTFIIRCLQ	1
AKT1	c.49G>A; p.E17K	1
ARAF	c.1702G>C; p.E568Q	1
ARID1A	c.1543C>T; p.Q515*	1
ARID1A	c.2147delC; p.P716fs*26	1
ARID1A	c.3175_3176insGAAACCTCTGGACCTCTATCGCCTCTATGTGTCTGTGA; p.K1059fs*13	1
ARID1A	c.3634C>T; p.Q1212*	1
ARID1A	c.4189C>T; p.Q1397*	1
ARID1A	c.5975C>G; p.S1992*	1
ARID1A	c.6291_6378del88; p.E2098fs*8	1
ARID1A	c.6415_6416insT; p.P2139fs*11	1
ARID1A	c.6749_6750delAG; p.E2250fs*27	1
ASXL1	c.4019_4026delCAAGCACA; p.P1340fs*28	1
ATM	c.1010G>A; p.R337H	1
ATM	c.3799G>T; p.E1267*	1
ATM	c.7015_7016delAG; p.R2339fs*33	1
ATM	c.8158G>C; p.D2720H	1
ATR	c.4153-258_4204del310; p.splice site 4153-258_4204del310	1
ATRX	c.3580A>T; p.K1194*	1
BCOR	c.3622C>T; p.Q1208*	1
BRCA2	c.1898delA; p.N633fs*11	1
BRCA2	c.9155G>A; p.R3052Q	1
BRD4	c.4021-1G>C; p.splice site 4021-1G>C	1
CASP8	c.1279_1280delGT; p.V427fs*28	1
CBFB	c.78+1G>A; p.splice site 78+1G>A	1
CBFB	c.97_110delAGGGACCGGCCCCA; p.D34fs*44	1
CDH1	c.1792C>T; p.R598*	1
CDH1	c.1921C>T; p.Q641*	1
CDH1	c.1955delT; p.L652fs*1	1
CDH1	c.1993delA; p.I665fs*14	1
CDH1	c.220C>T; p.R74*	1
CDH1	c.2460_2463delTGAC; p.D821fs*24	1
CDH1	c.67C>T; p.Q23*	2
CDH1	c.81delC; p.C28fs*28	1
CDH1	c.832+1G>T; p.splice site 832+1G>T	1
CDKN1B	c.340_347delCCGGCGGC; p.P114fs*8	1
CDKN2A	c.151-1G>A; p.splice site 151-1G>A	1
CHEK1	c.515C>A; p.P172Q	1
CHEK2	c.1100delC; p.T367fs*15	1
CHEK2	c.470T>C; p.I157T	3
CREBBP	c.4328_4332delGTTGC; p.R1443fs*8	1
CREBBP	c.86-1G>C; p.splice site 86-1G>C	1
CTNNA1	c.966_967insAAGCATCATTAGTGGGGCTGCCTTGATGGCCGACTCG; p.S323fs*4	1

Gene	HGVS mutation nomenclature	N
CTNNB1	c.1400_1413delCTCTTCGTCATCTG; p.A467fs*25	1
ERBB2	c.2089G>C; p.V697L	1
ERBB2	c.2264T>C; p.L755S	2
ERBB2	c.2325_2326insTACGTGATGGCT; p.A775_G776insYVMA	1
ERBB3	c.2783A>G; p.E928G	1
ERBB3	c.850G>C; p.G284R	1
ERBB4	c.908C>T; p.S303F	1
ERCC4	c.2395C>T; p.R799W	1
ESR1	c.1138G>C; p.E380Q	2
ESR1	c.1609T>A; p.Y537N	2
ESR1	c.1610A>C; p.Y537S	1
ESR1	c.1610A>G; p.Y537C	1
ESR1	c.1613A>G; p.D538G	3
EZH2	c.435C>G; p.F145L	1
FGFR2	c.1647T>A; p.N549K	1
GABRA6	c.682G>A; p.V228I	1
GATA3	c.1006_1007insG; p.D336fs*17	1
GATA3	c.1051-46_1164del160; p.splice site 1051-46_1164del160	1
GATA3	c.1089_1122del34; p.T364fs*30	1
GATA3	c.1211_1212insC; p.S405fs*103	1
GATA3	c.1223_1224insC; p.P409fs*99	1
GATA3	c.1224_1225insG; p.P409fs*99	2
GATA3	c.1239_1240insC; p.S414fs*94	1
GATA3	c.1263_1264insG; p.P422fs*86	1
GATA3	c.1266_1270delGATGC; p.M423fs*83	1
GATA3	c.1277_1278insCCGCC; p.S427fs*51	1
GATA3	c.1287_1288delGT; p.S430fs*77	1
GATA3	c.1315delA; p.M439fs*37	1
GATA3	c.881T>A; p.M294K	1
GATA3	c.988_989delAG; p.R330fs*22	1
GATA3	c.988_989insA; p.R330fs*23	1
GRM3	c.2273C>T; p.T758M	1
KDM5C	c.2293_2510del344; p.splice site 2293_2510del344	1
KDR	c.1860C>A; p.S620R	1
KEL	c.2T>C; p.M1T	1
KLHL6	c.821C>T; p.T274M	1
KRAS	c.34G>T; p.G12C	1
KRAS	c.35G>T; p.G12V	1
MAP2K1	c.607G>A; p.E203K	1
MAP2K4	c.1086+1G>A; p.splice site 1086+1G>A	1
MAP2K4	c.328C>T; p.R110*	1
MAP2K4	c.921T>A; p.Y307*	1
MAP3K1	c.1013_1014insA; p.Y338fs*1	1
MAP3K1	c.1331_1332insC; p.I445fs*8	1
MAP3K1	c.1486delA; p.R496fs*61	1
MAP3K1	c.2187delT; p.I729fs*10	1
MAP3K1	c.2242_2245delTCAA; p.S748fs*13	1
MAP3K1	c.3879_3883delAGAGA; p.E1293fs*14	1
MAP3K1	c.4050_4051insACACT; p.E1351fs*28	1
MAP3K1	c.4144C>T; p.Q1382*	1

Gene	HGVS mutation nomenclature	*N*
MAP3K1	c.4390-1G>C; p.splice site 4390-1G>C	1
MAP3K1	c.862C>T; p.R288*	1
MEN1	c.166_187del22; p.T56fs*56	1
MEN1	c.505delG; p.A169fs*21	1
MEN1	c.898_902delCGGCC; p.D302fs*18	1
MRE11A	c.1726C>T; p.R576*	1
MSH3	c.2436-1G>A; p.splice site 2436-1G>A	1
MUTYH	c.494A>G; p.Y165C	1
NF1	c.1660C>T; p.Q554*	1
NF1	c.4084C>T; p.R1362*	1
NF1	c.4195C>T; p.Q1399*	1
NF1	c.4543C>T; p.Q1515*	1
NF1	c.5902C>T; p.R1968*	1
NF1	c.6007-1G>A; p.splice site 6007-1G>A	1
NF1	c.7006G>A; p.A2336T	1
NF1	c.7591C>T; p.Q2531*	1
NFE2L2	c.242G>A; p.G81D	1
NOTCH1	c.4723G>C; p.V1575L	1
NOTCH1	c.7541_7542delCT; p.P2514fs*4	1
NOTCH1	c.7543G>T; p.E2515*	1
NOTCH2	c.3406C>T; p.Q1136*	1
PALB2	c.1424_1425insC; p.R476fs*11	1
PALB2	c.2257C>T; p.R753*	1
PIK3CA	c.1035T>A; p.N345K	3
PIK3CA	c.115G>A; p.E39K	1
PIK3CA	c.1252G>A; p.E418K	1
PIK3CA	c.1356_1382del27; p.L452_V461>F	1
PIK3CA	c.1357G>C; p.E453Q	1
PIK3CA	c.1624G>A; p.E542K	3
PIK3CA	c.1633G>A; p.E545K	8
PIK3CA	c.1634A>C; p.E545A	1
PIK3CA	c.311C>T; p.P104L	2
PIK3CA	c.3127A>G; p.M1043V	1
PIK3CA	c.3140A>G; p.H1047R	11
PIK3CA	c.3140A>T; p.H1047L	3
PIK3CA	c.3145G>C; p.G1049R	1
PIK3CA	c.320A>G; p.N107S	1
PIK3R1	c.1300-21_1304>G; p.splice site 1300-21_1304>G	1
PIK3R1	c.1323_1358del36; p.I442_N453del	1
PIK3R1	c.1325_1357del33; p.I442_Y452del	1
PIK3R1	c.1425+1G>A; p.splice site 1425+1G>A	1
PIK3R1	c.1724_1729delAGACGA; p.K575_T576del	1
PPP2R1A	c.767C>T; p.S256F	1
PTEN	c.203A>G; p.Y68C	1
PTEN	c.351_450del100; p.N117fs*3	1
PTEN	c.497_498insT; p.T167fs*13	1
PTEN	c.733C>T; p.Q245*	1
PTEN	c.763_770delGTAGAGTT; p.V255fs*40	1
PTEN	c.955_958delACTT; p.T319fs*1	1
RAD51D	c.694C>T; p.R232*	1

Gene	HGVS mutation nomenclature	*N*
RB1	c.2773_*34del49; p.K925fs*23	1
REL	c.1261C>T; p.R421C	1
RET	c.1799G>A; p.R600Q	1
RNF43	c.1820C>T; p.S607L	1
SDHB	c.151_152delAA; p.K51fs*11	1
SMAD4	c.1054G>A; p.G352R	1
SMARCB1	c.118C>T; p.R40*	1
SOX9	c.1079_1080insC; p.Q361fs*217	1
SPEN	c.10228_10229delAA; p.N3410fs*4	1
SPEN	c.10753C>T; p.Q3585*	1
SPEN	c.2227C>T; p.Q743*	1
SPEN	c.2761C>T; p.Q921*	1
SPEN	c.316_356del41; p.G106fs*11	1
SPEN	c.4111C>T; p.R1371*	1
SPEN	c.6932C>G; p.S2311*	1
SPEN	c.7499_7500delAG; p.E2500fs*26	1
SPOP	c.570A>C; p.L190F	1
STK11	c.592_597+5GCCGAGGTAGG>CCCGA; p.splice site 592_597+5GCCGAGGTAGG>CCCGA	1
TBX3	c.1416_1417delGC; p.L473fs*218	1
TBX3	c.1812delC; p.Y605fs*27	1
TBX3	c.1941_1942insTG; p.P648fs*242	1
TBX3	c.2144_2152AGGCGGCCA>TT; p.E715fs*172	1
TBX3	c.628_632ACCAA>T; p.T210fs*32	1
TBX3	c.635_637delACA; p.N212del	1
TET2	c.5473C>T; p.Q1825*	1
TNFAIP3	c.1428G>A; p.M476I	1
TNFAIP3	c.499G>T; p.E167*	1
TP53	c.1014_1015delCG; p.F338fs*8	1
TP53	c.1015G>T; p.E339*	1
TP53	c.151G>T; p.E51*	1
TP53	c.227_279del53; p.A76fs*55	1
TP53	c.267delC; p.S90fs*33	1
TP53	c.329G>C; p.R110P	1
TP53	c.329G>T; p.R110L	1
TP53	c.358A>G; p.K120E	1
TP53	c.376-41_402del68; p.splice site 376-41_402del68	1
TP53	c.379T>A; p.S127T	1
TP53	c.399_400insG; p.F134fs*15	1
TP53	c.434T>C; p.L145P	2
TP53	c.445_466del22; p.S149fs*14	1
TP53	c.455_456insC; p.P153fs*28	1
TP53	c.488A>C; p.Y163S	1
TP53	c.524G>A; p.R175H	3
TP53	c.529_546delCCCCACCATGAGCGCTGC; p.P177_C182del	1
TP53	c.535_536insC; p.H179fs*2	1
TP53	c.536A>G; p.H179R	1
TP53	c.574C>T; p.Q192*	2
TP53	c.584T>C; p.I195T	1
TP53	c.586C>T; p.R196*	1

**Gene**	**HGVS mutation nomenclature**	***N***
TP53	c.594_602delAGGAAATTT; p.G199_L201del	1
TP53	c.637C>T; p.R213*	1
TP53	c.665C>T; p.P222L	1
TP53	c.672+1G>T; p.splice site 672+1G>T	1
TP53	c.700T>C; p.Y234H	1
TP53	c.722C>T; p.S241F	1
TP53	c.742C>G; p.R248G	1
TP53	c.743G>A; p.R248Q	1
TP53	c.814G>A; p.V272M	1
TP53	c.838A>G; p.R280G	1
TP53	c.839G>A; p.R280K	1
TP53	c.839G>C; p.R280T	1
TP53	c.844C>T; p.R282W	1
TP53	c.892G>T; p.E298*	1
TP53	c.916C>T; p.R306*	1
TP53	c.960G>C; p.K320N	1
TP53	c.993+2T>G; p.splice site 993+2T>G	1
VHL	c.5_6insGCGGAGGGAATGCC; p.A5fs*14	1

Supplementary Table 7: List of all copy-number-alterations (CNA) from the somatic genotyping (*N*=94)

Gene	Copy number	CNA exons	CNA ratio	CNA type	CNA position
AKT1	7	13 of 13	1.75	amplification	chr14:105187919-105302907
AKT3	7	14 of 14	2.08	amplification	chr1:243619000-244056522
AKT3	7	14 of 14	1.53	amplification	chr1:243627223-244056522
AR	10	9 of 9	3.03	amplification	chrX:66764988-66943683
ARFRP1	7	6 of 6	2.28	amplification	chr20:62282005-62383121
ARFRP1	7	6 of 6	2.13	amplification	chr20:62282005-62383121
ARFRP1	6	6 of 6	2.20	amplification	chr20:62282005-62383121
ARFRP1	12	6 of 6	5.13	amplification	chr20:62282005-62383121
AURKA	7	8 of 8	2.13	amplification	chr20:54897975-55013001
AURKA	6	8 of 8	2.57	amplification	chr20:54897975-55013001
AURKA	6	8 of 8	2.45	amplification	chr20:54897975-55013001
AURKB	11	8 of 8	3.63	amplification	chr17:8059789-8161279
BCL2L1	7	4 of 4	2.16	amplification	chr20:30253751-30310021
BCL2L1	8	4 of 4	2.00	amplification	chr20:30253751-30310021
CCND1	23	5 of 5	8.00	amplification	chr11:69412013-69502020
CCND1	9	5 of 5	1.92	amplification	chr11:69412013-69502020
CCND1	10	5 of 5	2.23	amplification	chr11:69412013-69502020
CCND1	7	5 of 5	3.12	amplification	chr11:69449390-69490355
CCND1	16	5 of 5	4.41	amplification	chr11:69412013-69502020
CCND1	68	5 of 5	15.14	amplification	chr11:69412013-69502020
CCND1	7	5 of 5	3.46	amplification	chr11:69412013-69502020
CCND1	7	5 of 5	2.14	amplification	chr11:69412013-69490355
CCND1	14	5 of 5	4.69	amplification	chr11:69412013-69502020
CCND1	16	5 of 5	2.75	amplification	chr11:69412013-69502020
CCND1	8	5 of 5	3.43	amplification	chr11:69412013-69502020
CCND1	9	5 of 5	2.46	amplification	chr11:69442323-69502020
CCND1	11	5 of 5	2.23	amplification	chr11:69412013-69502020
CCND1	8	5 of 5	3.66	amplification	chr11:69412013-69502020
CCND1	32	5 of 5	4.66	amplification	chr11:69412013-69502020
CCND1	23	5 of 5	6.82	amplification	chr11:69412013-69502020
CCND1	45	5 of 5	13.09	amplification	chr11:69412013-69502020
CCND1	6	5 of 5	2.75	amplification	chr11:69412013-69502020
CCND1	12	5 of 5	4.56	amplification	chr11:69412013-69502020
CCND1	19	5 of 5	4.69	amplification	chr11:69412013-69502020
CCND1	53	5 of 5	8.82	amplification	chr11:69412013-69502020
CCND1	12	5 of 5	4.47	amplification	chr11:69431286-69502020
CCND1	19	5 of 5	4.56	amplification	chr11:69412013-69502020
CCND2	8	5 of 5	2.03	amplification	chr12:4335813-4445571
CCND3	8	5 of 5	1.74	amplification	chr6:41853880-41956362
CCNE1	7	10 of 10	1.60	amplification	chr19:30253918-30349126
CCNE1	21	10 of 10	6.28	amplification	chr19:30276384-30360638
CCNE1	6	10 of 10	2.10	amplification	chr19:30253918-30360638
CD274	8	7 of 7	1.96	amplification	chr9:5456074-5470632
CDK6	9	7 of 7	2.41	amplification	chr7:92205279-92504369
CDK8	6	12 of 13	2.53	amplification	chr13:26911676-27021216
CDKN2A	0	5 of 5	0.79	loss	chr9:21968169-21994453
CDKN2A	0	5 of 5	0.43	loss	chr9:21853212-21998002

Gene	Copy number	CNA exons	CNA ratio	CNA type	CNA position
CDKN2A	0	5 of 5	0.40	loss	chr9:21853212-21998002
CDKN2A	0	5 of 5	0.35	loss	chr9:21853212-21998002
CDKN2A	0	5 of 5	0.13	loss	chr9:21853212-21998002
CDKN2A	0	5 of 5	0.45	loss	chr9:21968169-21994453
CDKN2A	0	5 of 5	0.20	loss	chr9:21853212-21998002
CDKN2A	0	4 of 5	0.21	loss	chr9:21853212-21981592
CDKN2B	0	5 of 5	0.79	loss	chr9:22002170-22010784
CDKN2B	0	5 of 5	0.13	loss	chr9:21998748-22101832
CDKN2B	0	5 of 5	0.43	loss	chr9:21998748-22101832
CDKN2B	0	5 of 5	0.20	loss	chr9:21998748-22101832
CDKN2B	0	5 of 5	0.49	loss	chr9:21998748-22101832
CDKN2B	0	5 of 5	0.35	loss	chr9:21998748-22101832
CSF3R	7	16 of 16	2.46	amplification	chr1:36931684-36945130
CTNNA1	0	4 of 17	0.23	loss	chr5:138221876-138253587
CUL4A	7	20 of 20	1.91	amplification	chr13:113863941-113917913
EMSY	6	20 of 20	1.62	amplification	chr11:76120008-76310898
EMSY	9	20 of 20	2.83	amplification	chr11:76113654-76310898
EMSY	10	20 of 20	4.03	amplification	chr11:76113654-76310898
EMSY	43	20 of 20	7.31	amplification	chr11:76113654-76310898
EMSY	17	20 of 20	5.46	amplification	chr11:76113654-76310898
EMSY	45	20 of 20	9.85	amplification	chr11:76113654-76310898
EMSY	9	20 of 20	2.23	amplification	chr11:76120008-76310898
EMSY	7	20 of 20	3.29	amplification	chr11:76113654-76310898
EMSY	6	20 of 20	2.46	amplification	chr11:76142889-76310898
EMSY	8	20 of 20	1.66	amplification	chr11:76113654-76310898
EPHB4	10	17 of 17	2.08	amplification	chr7:100401082-100424691
ERBB2	6	27 of 27	1.55	amplification	chr17:37807689-37933832
ERBB2	6	27 of 27	1.66	amplification	chr17:37807689-37933832
ERBB2	7	27 of 27	2.03	amplification	chr17:37807689-37933832
ERBB2	7	27 of 27	1.85	amplification	chr17:37807689-37933832
ESR1	23	8 of 8	8.82	amplification	chr6:152090577-152469340
FAS	0	10 of 10	0.49	loss	chr10:90750583-90775607
FGF12	8	6 of 6	2.73	amplification	chr3:191820777-192487207
FGF19	20	3 of 3	5.86	amplification	chr11:69514029-69561786
FGF19	8	3 of 3	3.03	amplification	chr11:69514029-69561786
FGF19	7	3 of 3	2.48	amplification	chr11:69514029-69561786
FGF19	32	3 of 3	4.66	amplification	chr11:69514029-69561786
FGF19	6	3 of 3	2.60	amplification	chr11:69514029-69561786
FGF19	11	3 of 3	2.10	amplification	chr11:69514029-69561786
FGF19	15	3 of 3	3.78	amplification	chr11:69514029-69561786
FGF19	16	3 of 3	2.51	amplification	chr11:69514029-69518644
FGF19	6	3 of 3	1.57	amplification	chr11:69514029-69561786
FGF19	10	3 of 3	3.58	amplification	chr11:69514029-69561786
FGF19	10	3 of 3	1.99	amplification	chr11:69514029-69561786
FGF19	9	3 of 3	2.85	amplification	chr11:69514029-69561786
FGF19	9	3 of 3	1.78	amplification	chr11:69514029-69561786
FGF19	13	3 of 3	3.18	amplification	chr11:69514029-69536217
FGF19	8	3 of 3	3.27	amplification	chr11:69514029-69561786
FGF19	6	3 of 3	2.41	amplification	chr11:69514029-69561786
FGF19	7	3 of 3	2.64	amplification	chr11:69514029-69561786

Gene	Copy number	CNA exons	CNA ratio	CNA type	CNA position
FGF19	7	3 of 3	1.60	amplification	chr11:69514029-69561786
FGF19	53	3 of 3	12.38	amplification	chr11:69514029-69561786
FGF19	13	3 of 3	5.24	amplification	chr11:69514029-69561786
FGF19	39	3 of 3	6.96	amplification	chr11:69514029-69561786
FGF19	41	3 of 3	11.71	amplification	chr11:69514029-69561786
FGF23	8	3 of 3	2.10	amplification	chr12:4462589-4488748
FGF23	14	3 of 3	2.13	amplification	chr12:4462589-4488748
FGF3	8	3 of 3	3.46	amplification	chr11:69580315-69683592
FGF3	6	3 of 3	2.28	amplification	chr11:69580315-69683592
FGF3	9	3 of 3	1.78	amplification	chr11:69580315-69683592
FGF3	12	3 of 3	4.96	amplification	chr11:69580315-69683592
FGF3	12	3 of 3	4.44	amplification	chr11:69580315-69683592
FGF3	7	3 of 3	1.83	amplification	chr11:69580315-69683592
FGF3	47	3 of 3	11.00	amplification	chr11:69580315-69633701
FGF3	39	3 of 3	6.96	amplification	chr11:69580315-69683592
FGF3	9	3 of 3	2.07	amplification	chr11:69614838-69683592
FGF3	8	3 of 3	1.51	amplification	chr11:69580315-69683592
FGF3	10	3 of 3	2.14	amplification	chr11:69580315-69683592
FGF3	15	3 of 3	3.34	amplification	chr11:69580315-69683592
FGF3	11	3 of 3	2.23	amplification	chr11:69580315-69683592
FGF3	40	3 of 3	11.39	amplification	chr11:69580315-69683592
FGF3	6	3 of 3	2.30	amplification	chr11:69625072-69683592
FGF3	9	3 of 3	2.46	amplification	chr11:69580315-69666638
FGF3	20	3 of 3	5.78	amplification	chr11:69580315-69683592
FGF3	7	3 of 3	2.48	amplification	chr11:69580315-69683592
FGF3	6	3 of 3	2.22	amplification	chr11:69580315-69683592
FGF3	7	3 of 3	1.62	amplification	chr11:69580315-69683592
FGF3	47	3 of 3	6.45	amplification	chr11:69580315-69683592
FGF4	20	3 of 3	5.66	amplification	chr11:69588076-69589852
FGF4	7	3 of 3	2.60	amplification	chr11:69588076-69589852
FGF4	8	3 of 3	1.48	amplification	chr11:69588076-69589852
FGF4	8	3 of 3	3.66	amplification	chr11:69588076-69589852
FGF4	7	3 of 3	1.83	amplification	chr11:69588076-69589852
FGF4	12	3 of 3	4.44	amplification	chr11:69588076-69589852
FGF4	47	3 of 3	6.50	amplification	chr11:69588076-69589852
FGF4	7	3 of 3	2.51	amplification	chr11:69588076-69589852
FGF4	15	3 of 3	3.23	amplification	chr11:69588076-69589852
FGF4	38	3 of 3	11.00	amplification	chr11:69588076-69589852
FGF4	6	3 of 3	1.57	amplification	chr11:69588076-69589852
FGF4	9	3 of 3	2.64	amplification	chr11:69588076-69589852
FGF4	11	3 of 3	2.85	amplification	chr11:69588076-69589852
FGF4	10	3 of 3	2.35	amplification	chr11:69588076-69589852
FGF4	8	3 of 3	3.14	amplification	chr11:69588076-69589852
FGF4	6	3 of 3	2.10	amplification	chr11:69588076-69589852
FGF4	11	3 of 3	4.56	amplification	chr11:69588076-69589852
FGF4	44	3 of 3	10.27	amplification	chr11:69588076-69589852
FGF4	42	3 of 3	7.46	amplification	chr11:69588076-69589852
FGF4	6	3 of 3	2.39	amplification	chr11:69588076-69589852
FGF4	9	3 of 3	1.92	amplification	chr11:69588076-69589852
FGF6	8	3 of 3	2.04	amplification	chr12:4494593-4598143

Gene	Copy number	CNA exons	CNA ratio	CNA type	CNA position
FGF6	14	3 of 3	2.03	amplification	chr12:4494593-4593430
FGFR1	12	18 of 18	3.07	amplification	chr8:38271145-38322355
FGFR1	89	18 of 18	14.93	amplification	chr8:38226104-38363070
FGFR1	7	18 of 18	1.83	amplification	chr8:38231865-38363070
FGFR1	17	18 of 18	3.39	amplification	chr8:38226104-38363070
FGFR1	9	18 of 18	3.27	amplification	chr8:38226104-38363070
FGFR1	25	18 of 18	5.70	amplification	chr8:38226104-38363070
FGFR1	36	18 of 18	9.92	amplification	chr8:38226104-38363070
FGFR1	34	18 of 18	4.86	amplification	chr8:38226104-38363070
FGFR1	8	18 of 18	3.05	amplification	chr8:38271145-38363070
FGFR1	6	18 of 18	2.06	amplification	chr8:38226104-38363070
FGFR1	9	18 of 18	2.28	amplification	chr8:38271145-38363070
FGFR1	11	18 of 18	1.45	amplification	chr8:38271145-38363070
FGFR1	15	18 of 18	2.28	amplification	chr8:38226104-38363070
FGFR1	17	18 of 18	2.83	amplification	chr8:38259351-38363070
FGFR1	8	18 of 18	2.14	amplification	chr8:38231865-38363070
FGFR1	16	18 of 18	4.29	amplification	chr8:38226104-38363070
FGFR4	6	16 of 16	2.66	amplification	chr5:176449475-176569318
FGFR4	8	16 of 16	2.23	amplification	chr5:176449475-176569318
GNAS	11	15 of 15	4.32	amplification	chr20:57415161-57485884
GNAS	7	15 of 15	2.36	amplification	chr20:57415161-57485884
GNAS	6	13 of 15	2.41	amplification	chr20:57466781-57485884
GNAS	7	13 of 15	2.17	amplification	chr20:57466781-57485884
HGF	7	19 of 19	1.83	amplification	chr7:81331896-81399308
HGF	11	19 of 19	2.51	amplification	chr7:81287783-81435614
HRAS	9	5 of 5	2.41	amplification	chr11:511507-584252
IGF1R	13	20 of 21	3.73	amplification	chr15:99192792-99491937
IKBKE	58	20 of 20	13.55	amplification	chr1:206646549-206669525
IKBKE	8	20 of 20	1.77	amplification	chr1:206646549-206669525
IRS2	15	5 of 5	4.14	amplification	chr13:110406186-110438400
IRS2	75	5 of 5	17.51	amplification	chr13:110406186-110438400
JAK2	8	23 of 23	2.08	amplification	chr9:5021987-5126801
JUN	7	3 of 3	2.91	amplification	chr1:59219094-59298520
KDM5A	11	28 of 28	3.56	amplification	chr12:394621-498257
KDM5A	8	28 of 28	2.17	amplification	chr12:394621-498257
KDM5A	8	28 of 28	2.08	amplification	chr12:394621-498257
KDM5A	7	28 of 28	2.91	amplification	chr12:394621-498257
KRAS	8	5 of 5	1.88	amplification	chr12:25344730-25417157
KRAS	8	5 of 5	2.06	amplification	chr12:25344730-25448217
LYN	9	12 of 12	2.60	amplification	chr8:56854418-56922669
LYN	8	12 of 12	1.96	amplification	chr8:56854418-56922669
LYN	10	12 of 12	1.89	amplification	chr8:56854418-56922669
LYN	11	12 of 12	2.75	amplification	chr8:56854418-56922669
LYN	7	12 of 12	2.06	amplification	chr8:56854418-56922669
LYN	10	12 of 12	2.20	amplification	chr8:56854418-56922669
MAP2K1	7	11 of 11	1.99	amplification	chr15:66679660-66782960
MAP2K4	0	12 of 12	0.49	loss	chr17:11835312-12105692
MAP2K4	0	8 of 12	0.18	loss	chr17:11835312-12016677
MAP2K4	0	10 of 12	0.41	loss	chr17:11958192-12105692
MAP2K4	0	4 of 12	0.21	loss	chr17:12028584-12105692

Gene	Copy number	CNA exons	CNA ratio	CNA type	CNA position
MAP2K4	0	8 of 12	0.47	loss	chr17:11995871-12105692
MAP2K4	0	5 of 12	0.40	loss	chr17:11835312-12006910
MAP2K4	0	12 of 12	0.32	loss	chr17:11835312-12105692
MCL1	8	5 of 5	2.95	amplification	chr1:150518281-150600810
MCL1	7	5 of 5	2.46	amplification	chr1:150511135-150600810
MCL1	8	5 of 5	2.08	amplification	chr1:150511135-150552548
MCL1	7	5 of 5	2.55	amplification	chr1:150511135-150600810
MDM2	10	11 of 11	2.33	amplification	chr12:69153996-69277205
MDM2	12	11 of 11	3.73	amplification	chr12:69153996-69277205
MDM2	26	11 of 11	6.23	amplification	chr12:69153996-69277205
MDM2	6	11 of 11	2.17	amplification	chr12:69202199-69277205
MDM4	8	10 of 10	1.84	amplification	chr1:204494620-204518810
MDM4	7	8 of 10	1.95	amplification	chr1:204494620-204513812
MDM4	7	10 of 10	2.10	amplification	chr1:204494620-204518810
MDM4	8	10 of 10	2.07	amplification	chr1:204494620-204518810
MDM4	8	9 of 10	1.87	amplification	chr1:204495460-204518810
MTAP	0	7 of 8	0.39	loss	chr9:21815410-21862058
MTAP	0	7 of 8	0.77	loss	chr9:21815410-21862058
MTAP	0	8 of 8	0.18	loss	chr9:21802699-21862058
MTAP	0	8 of 8	0.13	loss	chr9:21802699-21862058
MTAP	0	4 of 8	0.22	loss	chr9:21837893-21862058
MTAP	0	8 of 8	0.42	loss	chr9:21802699-21862058
MTAP	0	8 of 8	0.36	loss	chr9:21802699-21862058
MTAP	0	7 of 8	0.57	loss	chr9:21815410-21862058
MYC	11	5 of 5	4.59	amplification	chr8:128706589-128801451
MYC	19	5 of 5	4.59	amplification	chr8:128706589-128801451
MYC	8	5 of 5	2.00	amplification	chr8:128719852-128801451
MYC	8	5 of 5	2.35	amplification	chr8:128742842-128772066
MYC	8	5 of 5	2.20	amplification	chr8:128706589-128801451
MYC	7	5 of 5	2.46	amplification	chr8:128706589-128801451
MYC	39	5 of 5	10.78	amplification	chr8:128706589-128801451
MYC	7	5 of 5	1.59	amplification	chr8:128706589-128801451
MYC	13	5 of 5	4.03	amplification	chr8:128706589-128801451
MYC	8	5 of 5	2.58	amplification	chr8:128706589-128772066
MYC	7	5 of 5	3.16	amplification	chr8:128706589-128801451
MYC	15	5 of 5	2.97	amplification	chr8:128706589-128801451
MYC	8	5 of 5	1.69	amplification	chr8:128706589-128801451
MYC	7	5 of 5	2.08	amplification	chr8:128727876-128753204
MYC	8	5 of 5	2.43	amplification	chr8:128706589-128780331
MYC	7	4 of 5	1.87	amplification	chr8:128706589-128751994
MYC	6	5 of 5	1.71	amplification	chr8:128706589-128801451
MYC	14	5 of 5	3.14	amplification	chr8:128706589-128772066
MYCL1	14	5 of 5	5.78	amplification	chr1:40360900-40368610
NF1	0	36 of 59	0.26	loss	chr17:29422292-29596265
NFKBIA	7	6 of 6	2.85	amplification	chr14:35871177-35873850
NOTCH3	7	33 of 33	1.68	amplification	chr19:15271472-15311722
PARP1	9	23 of 23	2.35	amplification	chr1:226549136-226595635
PDCD1LG2	8	6 of 6	1.75	amplification	chr9:5522509-5570021
PIK3C2B	7	32 of 32	2.60	amplification	chr1:204393979-204429102
PIK3C2B	7	32 of 32	2.30	amplification	chr1:204393979-204438930

Gene	Copy number	CNA exons	CNA ratio	CNA type	CNA position
PIK3C2B	8	32 of 32	2.16	amplification	chr1:204393979-204438930
PIK3C2B	8	32 of 32	1.80	amplification	chr1:204393979-204438930
PIK3C2B	8	26 of 32	2.46	amplification	chr1:204393979-204429102
PIK3C2B	11	32 of 32	2.31	amplification	chr1:204393979-204438930
PIK3C2B	7	32 of 32	1.85	amplification	chr1:204393979-204438930
PIK3CA	55	20 of 20	12.82	amplification	chr3:178875270-178996445
PIK3CA	7	20 of 20	1.61	amplification	chr3:178875270-178969237
PRKCI	7	18 of 18	1.66	amplification	chr3:169940443-170020936
PTEN	0	9 of 9	0.49	loss	chr10:89548329-89795653
PTEN	0	9 of 9	0.54	loss	chr10:89548329-89795653
PTEN	0	9 of 9	0.34	loss	chr10:89599345-89725229
PTEN	0	9 of 9	0.30	loss	chr10:89548329-89764620
PTEN	0	1 of 9	0.23	loss	chr10:89653759-89653888
RAD21	11	13 of 13	2.16	amplification	chr8:117859738-117878968
RAD21	11	13 of 13	2.89	amplification	chr8:117859738-117878968
RAD21	10	13 of 13	3.01	amplification	chr8:117859738-117878968
RAD21	8	13 of 13	2.30	amplification	chr8:117859738-117878968
RAD21	8	13 of 13	2.38	amplification	chr8:117859738-117878968
RAD21	9	13 of 13	4.44	amplification	chr8:117859738-117878968
RAD21	8	13 of 13	2.85	amplification	chr8:117859738-117878968
RAD21	9	13 of 13	2.41	amplification	chr8:117859738-117878968
RAD21	7	13 of 13	1.93	amplification	chr8:117859738-117878968
RAD21	15	13 of 13	4.38	amplification	chr8:117859738-117878968
RAD21	16	13 of 13	6.82	amplification	chr8:117859738-117878968
RAD21	8	13 of 13	1.84	amplification	chr8:117859738-117878968
RAD21	7	13 of 13	2.46	amplification	chr8:117859738-117878968
RAD21	7	13 of 13	1.93	amplification	chr8:117859738-117878968
RAD21	11	13 of 13	3.16	amplification	chr8:117859738-117878968
RAD21	10	13 of 13	2.58	amplification	chr8:117859738-117878968
RAD21	11	12 of 13	1.97	amplification	chr8:117861161-117878968
RAD21	13	13 of 13	2.97	amplification	chr8:117859738-117878968
RAD21	8	13 of 13	2.11	amplification	chr8:117859738-117878968
RAD21	9	13 of 13	2.75	amplification	chr8:117859738-117878968
RAD21	9	13 of 13	2.81	amplification	chr8:117859738-117878968
RAD21	26	13 of 13	10.13	amplification	chr8:117859738-117878968
RAD21	9	13 of 13	1.80	amplification	chr8:117859738-117878968
RAF1	8	16 of 16	2.33	amplification	chr3:12576837-12709494
RB1	0	6 of 27	0.60	loss	chr13:48947519-48972066
RICTOR	6	39 of 39	2.22	amplification	chr5:38942378-39074519
RICTOR	8	39 of 39	2.10	amplification	chr5:38942378-39074519
ROS1	8	43 of 43	3.29	amplification	chr6:117609654-117746822
STK11	0	8 of 9	0.24	loss	chr19:1120199-1223171
TBX3	0	8 of 8	0.20	loss	chr12:115109645-115121005
TERC	7	3 of 3	2.45	amplification	chr3:169482000-169483150
TERC	7	3 of 3	2.04	amplification	chr3:169482000-169483150
TP53	0	14 of 14	0.12	loss	chr17:7537783-7580715
VEGFA	8	8 of 8	2.71	amplification	chr6:43738443-43752419
VEGFA	8	8 of 8	1.62	amplification	chr6:43738443-43752419
WHSC1L1	14	23 of 23	3.73	amplification	chr8:38133159-38205689
WHSC1L1	8	23 of 23	1.77	amplification	chr8:38133159-38205689

Gene	Copy number	CNA exons	CNA ratio	CNA type	CNA position
WHSC1L1	9	23 of 23	3.66	amplification	chr8:38133159-38205689
WHSC1L1	9	23 of 23	2.17	amplification	chr8:38133159-38205689
WHSC1L1	110	23 of 23	18.51	amplification	chr8:38133159-38205689
WHSC1L1	19	23 of 23	4.59	amplification	chr8:38133159-38205689
WHSC1L1	15	23 of 23	2.79	amplification	chr8:38133159-38205689
WHSC1L1	6	23 of 23	2.36	amplification	chr8:38133159-38205689
WHSC1L1	21	23 of 23	4.44	amplification	chr8:38133159-38205689
WHSC1L1	45	23 of 23	11.63	amplification	chr8:38133159-38205689
WHSC1L1	16	23 of 23	1.99	amplification	chr8:38133159-38205689
WHSC1L1	25	23 of 23	6.96	amplification	chr8:38133159-38205689
WHSC1L1	10	23 of 23	3.81	amplification	chr8:38133159-38205689
WHSC1L1	10	23 of 23	2.62	amplification	chr8:38133159-38205689
WHSC1L1	45	23 of 23	5.98	amplification	chr8:38133159-38205689
WHSC1L1	21	23 of 23	3.41	amplification	chr8:38133159-38205689
WHSC1L1	33	23 of 23	7.36	amplification	chr8:38133159-38205689
ZNF217	7	4 of 4	2.03	amplification	chr20:52188273-52199365
ZNF217	16	4 of 4	4.32	amplification	chr20:52188273-52199365
ZNF217	18	4 of 4	7.36	amplification	chr20:52188273-52199365
ZNF217	8	4 of 4	4.14	amplification	chr20:52188273-52199365
ZNF217	6	4 of 4	2.71	amplification	chr20:52188273-52199365
ZNF703	6	2 of 2	2.33	amplification	chr8:37553497-37556192
ZNF703	15	2 of 2	2.55	amplification	chr8:37553497-37556192
ZNF703	129	2 of 2	21.86	amplification	chr8:37553497-37556192
ZNF703	20	2 of 2	4.96	amplification	chr8:37553497-37556192
ZNF703	9	2 of 2	4.92	amplification	chr8:37553497-37556192
ZNF703	14	2 of 2	3.68	amplification	chr8:37553497-37556192
ZNF703	26	2 of 2	6.11	amplification	chr8:37553497-37556192
ZNF703	8	2 of 2	2.13	amplification	chr8:37553497-37556192
ZNF703	42	2 of 2	11.47	amplification	chr8:37553497-37556192
ZNF703	9	2 of 2	2.53	amplification	chr8:37553497-37556192
ZNF703	15	2 of 2	5.43	amplification	chr8:37553497-37556192
ZNF703	30	2 of 2	8.51	amplification	chr8:37553497-37556192
ZNF703	14	2 of 2	3.01	amplification	chr8:37553497-37556192
ZNF703	15	2 of 2	6.06	amplification	chr8:37553497-37556192
ZNF703	27	2 of 2	3.97	amplification	chr8:37553497-37556192
ZNF703	27	2 of 2	5.82	amplification	chr8:37553497-37556192

Supplementary Table 8: List of all rearrangements from the somatic genotyping (*N*=94) [N/A: not applicable]

Gene 1	Gene 2	Rearrangement position 1	Rearrangement position 2
BRAF	N/A	chr7:140486583-140486699	chr7:139116608-139116734
TP53	*TP53*	chr17:7578183-7578433	chr17:7574147-7574262
BRIP1	N/A	chr17:59876577-59876803	chr17:59748563-59748968
NF1	*RAD23B*	chr17:29541541-29541743	chr9:110050850-110050993
FAM123B	N/A	chrX:63412747-63412867	chrX:63386047-63386285
APC	*EFCAB7*	chr5:112154644-112154920	chr1:64009672-64009843
KDM5C	N/A	chrX:53228051-53228203	chr5:1115976-1116248
EZH2	*CUL1*	chr7:148507454-148507658	chr7:148453200-148453394
NF1	*LAD1*	chr17:29482871-29483024	chr1:201355635-201355971
RB1	*RB1*	chr13:48942615-48942884	chr13:48957136-48957380
TNFRSF14	N/A	chr1:2491981-2492192	chr1:2438306-2438469
BRAF	N/A	chr7:140487094-140487355	chr7:140427718-140428130
BRIP1	N/A	chr17:59793179-59793451	chr17:56119950-56120291
NOTCH2	*DR1*	chr1:120465170-120465418	chr1:93818791-93819344
MYB	N/A	chr6:135527962-135528410	chr6:135568285-135568731
PTEN	*PTEN*	chr10:89711727-89711960	chr10:89679375-89679746
ETV6	N/A	chr12:12031262-12031542	chr12:12201782-12202021
NOTCH2	*SRCAP*	chr1:120464714-120464993	chr16:30710944-30711255
APC	N/A	chr5:112173653-112173871	chr14:91992638-91992980
FUBP1	N/A	chr1:78430609-78430804	chrX:135015488-135015680
SMARCA4	*ASNA1*	chr19:11134046-11134237	chr19:12852577-12852786
NBN	*XKR4*	chr8:90990392-90990636	chr8:56088404-56088587
CDH1	*CDH1*	chr16:68843969-68844194	chr16:68833685-68833854

Supplementary Table 9: Categorization of genes in the 26-gene testing panel

No	Gene Name	BRCA1/2	Other Homologous Recombination (HRR) Genes	Other DNA Repair Genes	Other BC Risk Genes
1	*APC*				X
2	*ATM*		X		
3	*BARD1*		X		
4	*BRCA1*	X			
5	*BRCA2*	X			
6	*BRIP1*			X	
7	*CDH1*				X
8	*CDKN2A*				X
9	*CHEK2*			X	
10	*FANCC*		X		
11	*KRAS*				X
12	*MEN1*				X
13	*MLH1*			X	
14	*MRE11A*			X	
15	*MSH2*			X	
16	*MSH6*			X	
17	*MUTHY*			X	
18	*NBN*			X	
19	*NF1*				X
20	*PALB2*		X		
21	*PMS2*			X	
22	*PTEN*				X
23	*RAD51C*		X		
24	*RAD51D*		X		
25	*TP53*				X
26	*XRCC2*		X		

Supplementary Table 10: Data categories captured in the PRAEGNANT study [ECOG: Eastern Cooperative Oncology Group - performance status; PRO: patient-reported outcome]

Data continuously captured if applicable	Data assessed at study entry	Data assessed at follow-up care appointments
Concomitant diseases	Life status, ECOG	Life status, ECOG
Concomitant medication	Quality of life	Quality of life
Cancer systemic therapies	Breast cancer risk factor questionnaire	Breast and axilla evaluation
Cancer radiotherapy	Breast and axilla evaluation	Distant metastasis evaluation
Cancer surgery	Distant metastasis evaluation	Biomaterial ascertainment
Breast cancer right side	Biomaterial ascertainment	PRO questionnaires
Breast cancer left side	PRO questionnaires	

Supplementary Table 11: List of genes included in the QIAseq panel

HGNC symbol	Reference Sequence	Ensemble Transcript Id	Chromosome	Start position	End position	strand	Ensemble gene id
APC	NM000038.5	ENST00000257430	5	112043195	112181936	1	ENSG00000134982
ATM	NM000051.3	ENST00000278616	11	108093211	108239829	1	ENSG00000149311
BARD1	NM000465.3	ENST00000260947	2	215590370	215674428	-1	ENSG00000138376
BRCA1	NM007294.3	ENST00000357654	17	41196312	41277500	-1	ENSG00000012048
BRCA2	NM000059.3	ENST00000544455	13	32889611	32973805	1	ENSG00000139618
BRIP1	NM032043.2	ENST00000259008	17	59758627	59940882	-1	ENSG00000136492
CDH1	NM004360.4	ENST00000261769	16	68771128	68869451	1	ENSG00000039068
CDKN2A	NM000077.4	ENST00000304494	9	21967751	21995300	-1	ENSG00000147889
CHEK2	NM007194.3	ENST00000328354	22	29083731	29138410	-1	ENSG00000183765
FANCC	NM000136.2	ENST00000289081	9	97861336	98079991	-1	ENSG00000158169
KRAS	NM004985.4	ENST00000311936	12	25357723	25403870	-1	ENSG00000133703
MEN1	NM130799.2	ENST00000312049	11	64570982	64578766	-1	ENSG00000133895
MLH1	NM000249.3	ENST00000231790	3	37034823	37107380	1	ENSG00000076242
MRE11A	NM005591.3	ENST00000323929	11	94152895	94227074	-1	ENSG00000020922
MSH2	NM000251.2	ENST00000233146	2	47630108	47789450	1	ENSG00000095002
MSH6	NM000179.2	ENST00000234420	2	47922669	48037240	1	ENSG00000116062
MUTYH	NM001128425.1	ENST00000450313	1	45794835	45806142	-1	ENSG00000132781
NBN	NM002485.4	ENST00000265433	8	90945564	91015456	-1	ENSG00000104320
NF1	NM001042492.2	ENST00000358273	17	29421945	29709134	1	ENSG00000196712
PALB2	NM024675.3	ENST00000261584	16	23614488	23652631	-1	ENSG00000083093
PMS2	NM000535.6	ENST00000265849	7	6012870	6048756	-1	ENSG00000122512
PTEN	NM000314.6	ENST00000371953	10	89622870	89731687	1	ENSG00000171862
RAD51C	NM058216.2	ENST00000337432	17	56769934	56811703	1	ENSG00000108384
RAD51D	NM001142571	ENST00000345365	17	33426811	33448541	-1	ENSG00000185379
TP53	NM000546.5	ENST00000269305	17	7565097	7590856	-1	ENSG00000141510
XRCC2	NM005431.1	ENST00000359321	7	152341864	152373250	-1	ENSG00000196584

Supplementary Figures

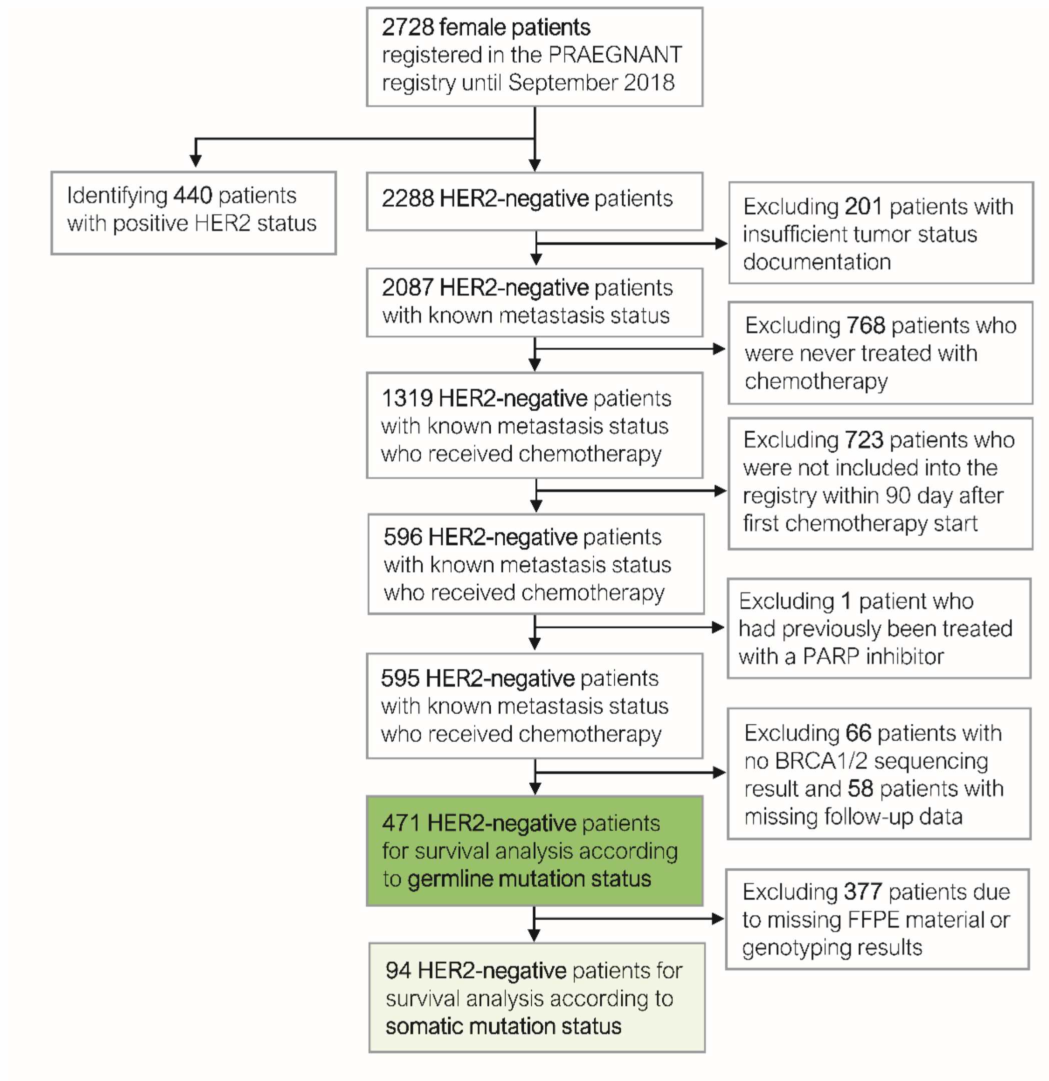

Supplementary Figure 1: Patient Flow Chart

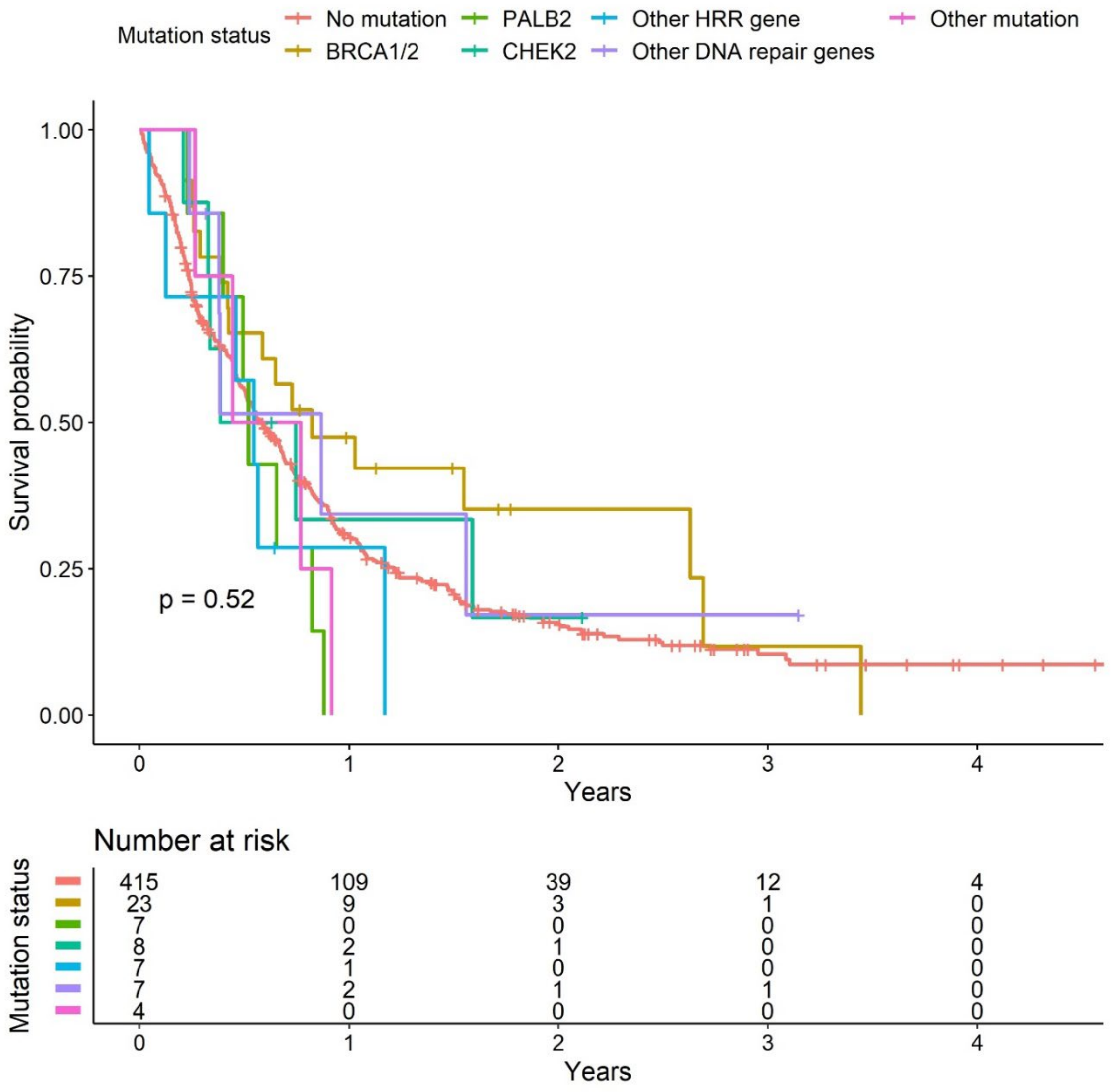

Supplementary Figure 2: Kaplan-Meier curves for progression free survival according to mutation status (7 groups)

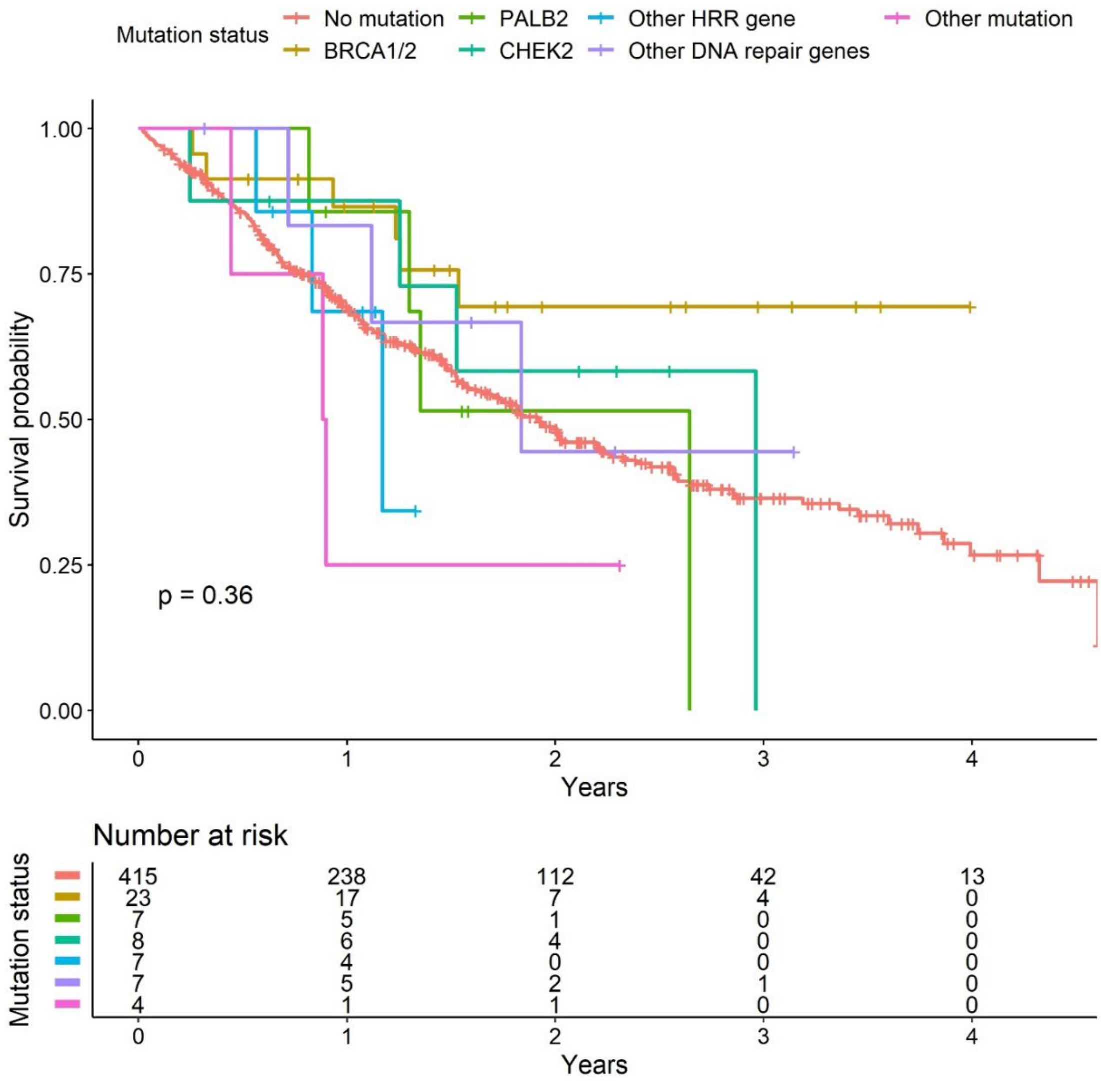

Supplementary Figure 3: Kaplan-Meier curves for overall survival according to mutation status (7 groups)
